# Robust Revascularization in Models of Limb Ischemia Using a Clinically Translatable Human Stem Cell-Derived Endothelial Cell Product

**DOI:** 10.1016/j.ymthe.2018.03.017

**Published:** 2018-03-28

**Authors:** Mark G. MacAskill, Jaimy Saif, Alison Condie, Maurits A. Jansen, Thomas J. MacGillivray, Adriana A.S. Tavares, Lucija Fleisinger, Helen L. Spencer, Marie Besnier, Ernesto Martin, Giovanni Biglino, David E. Newby, Patrick W.F. Hadoke, Joanne C. Mountford, Costanza Emanueli, Andrew H. Baker

**Affiliations:** 1 University/BHF Centre for Cardiovascular Science, University of Edinburgh, Edinburgh, UK; 2 Experimental Cardiovascular Medicine Division, Bristol Heart Institute, University of Bristol, Bristol, UK; 3 Scottish National Blood Transfusion Service, Edinburgh, UK; 4 Edinburgh Imaging, University of Edinburgh, Edinburgh, UK; 5 Institute of Cardiovascular and Medical Sciences, University of Glasgow, Glasgow, UK; 6 National Heart and Lung Institute, Imperial College London, London, UK

**Keywords:** cell therapy, critical limb ischemia, GMP

## Abstract

Pluripotent stem cell-derived differentiated endothelial cells offer high potential in regenerative medicine in the cardiovascular system. With the aim of translating the use of a human stem cell-derived endothelial cell product (hESC-ECP) for treatment of critical limb ischemia (CLI) in man, we report a good manufacturing practice (GMP)-compatible protocol and detailed cell tracking and efficacy data in multiple preclinical models. The clinical-grade cell line RC11 was used to generate hESC-ECP, which was identified as mostly endothelial (60% CD31^+^/CD144^+^), with the remainder of the subset expressing various pericyte/mesenchymal stem cell markers. Cell tracking using MRI, PET, and qPCR in a murine model of limb ischemia demonstrated that hESC-ECP was detectable up to day 7 following injection. Efficacy in several murine models of limb ischemia (immunocompromised/immunocompetent mice and mice with either type I/II diabetes mellitus) demonstrated significantly increased blood perfusion and capillary density. Overall, we demonstrate a GMP-compatible hESC-ECP that improved ischemic limb perfusion and increased local angiogenesis without engraftment, paving the way for translation of this therapy.

## Teaser

To translate hESC-ECP into the clinic for CLI treatment, MacAskill et al. developed a robust, GMP-compatible method for hESC-ECP generation. hESC-ECP remained within ischemic limbs for ∼7 days and significantly improved foot perfusion and capillary density in murine models of limb ischemia with/without an intact immune system or diabetes mellitus.

## Introduction

Peripheral arterial disease (PAD) is a common disorder and a major cause of morbidity and mortality, with 202 million people living with PAD globally in 2010.^1^ The most severely affected patients suffer from critical limb ischemia (CLI), characterized by rest pain, ulcerations, and/or gangrene, and have a very poor prognosis, with high rates of limb amputation and mortality.^2^ The situation is exacerbated in diabetes mellitus, which is one of the strongest risk factors for PAD. PAD is often asymptomatic in patients with diabetes mellitus due to peripheral neuropathy; thus, they may present later with more severe disease and an increased risk of amputation.^3^ Despite improvements in medical and surgical therapies, a significant portion of patients with CLI are considered “no option” for revascularization, and no medical therapy capable of reducing the need for amputation exists.^4^ Therefore, novel therapies that promote tissue regeneration and stimulation of angiogenesis are urgently needed for the treatment of CLI. Pro-angiogenic, cell-based therapies have significant potential in the treatment of ischemic disease but have not yet showed a clear clinical success, with the majority of CLI clinical trials carried out to date utilizing autologous bone marrow- or peripheral blood-derived cells in small pilot trials.^5^, ^6^ A recent meta-analysis of randomized controlled trials (RCTs) (16 RCTs, involving 774 patients) demonstrated that cell therapy in CLI is associated with reduced risk of major amputation.^7^ However, following reanalysis using placebo-controlled RCTs, these benefits were no longer significant. This calls for the need to test for alternative sources of stem cells, expanding to allogenic approaches. The efficacy of cells generated from pluripotent stem cells, such as human embryonic stem cell (hESC)-derived endothelial cells (ECs), has yet to be assessed in the clinic. Several preclinical studies assessing hESC-EC in murine models of CLI have demonstrated significant improvements in foot perfusion, accompanied, and hence possibly partially mediated, by increases in capillary density within ischemic limbs.^8^, ^9^, ^10^, ^11^, ^12^ Significantly, a breakthrough in the cardiovascular cell therapy field was made in a study that demonstrated the ability of hESC-cardiomyocytes to substantially engraft and regenerate infarcted non-human primate hearts,^13^ suggesting powerful regenerative effects of hESC-derived products. Therefore, a major focus of this field is to translate the use of pluripotent cell therapy into the clinic to assess its full potential.

A number of multi-step monolayer protocols have been shown to generate ECs from human pluripotent stem cells (hPSCs). Whereas these are excellent tools for investigating differentiation and development of EC function, the efficiency of conversion and reliance on xenobiotic reagents limits their clinical application. Even the most well-defined culture systems require use of animal products, such as Matrigel and/or fetal bovine serum (FBS), at different stages,^14^, ^15^, ^16^, ^17^ which are not appropriate for clinical translation. The source and grade of the starting PSC population is also important. Very few studies have used clinical-grade cells, with research-grade hESC and human-induced PSC (hiPSC) lines commonly utilized.^14^, ^15^, ^16^ To overcome these hurdles, we have adapted an appropriate protocol from the Cowan lab^14^ to provide compatibility for good manufacturing practice (GMP) and clinical-grade production, starting with the use of a clinical-grade cell line, RC11.^18^

We propose that administration of the hESC-EC product (ECP) derived using this protocol would be suitable for treatment of patients with CLI by stimulating angiogenesis in the affected limb. The final cell product, rather than purified ECs, was used, as it was considered that a further cell-sorting step would hinder GMP-compatible production, and it is possible that multiple cell populations contribute to the beneficial effect of cell administration. The aims of this investigation were to translate the use of hESC-ECP therapy to the clinic for CLI by (1) developing a robust, clinical-grade, and GMP-compatible protocol for the generation of hESC-ECP and characterization of the final cell product; (2) assessing the distribution of hESC-ECP post-transplantation using detailed imaging studies; and (3) evaluating the efficacy of hESC-ECP in a series of complex models using immunosuppressed/immunocompetent animals, mice with type I or type II diabetes mellitus, and transplant of cells either immediately after ischemia induction (acute ischemia) or after 3 days (established ischemia).

## Results

### Endothelial Differentiation of Clinical-Grade hESCs Using GMP-Compatible Reagents

**Figure 1:**
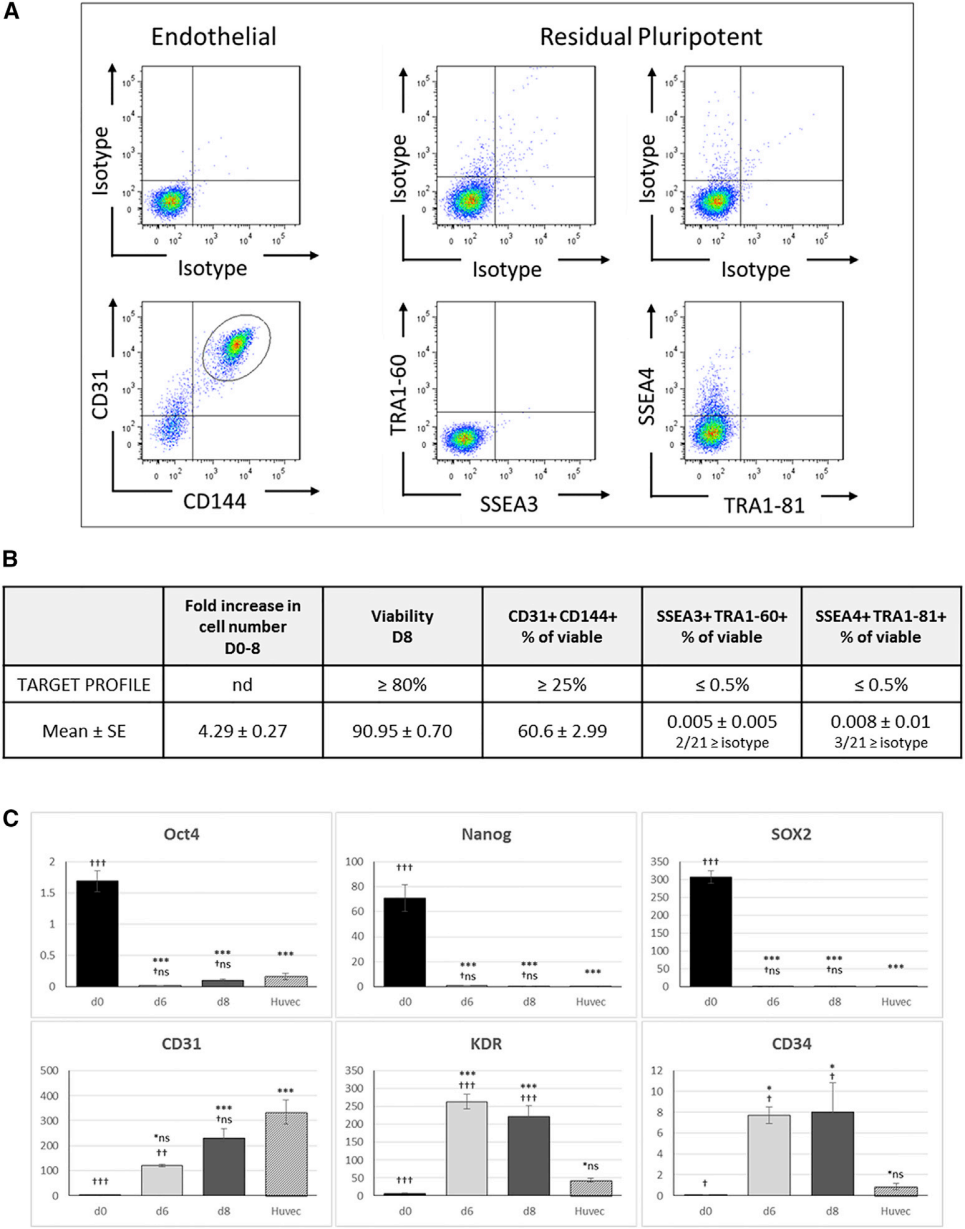
Endothelial Differentiation of the Clinical-Grade hESC Line RC11 Differentiated cells analyzed on day 8 of the protocol predominantly co-expressed the endothelial markers CD31 and CD144 with few, if any, detectable residual pluripotent hESCs. (A) Representative flow cytometric analysis for the endothelial (left panels) and pluripotent markers (middle and right panels) with the appropriate isotype controls is shown. Cells were pre-gated for viable cells (FSC/SSC; 10,000 events) and doublet exclusion (FSC-A/FSC-H). (B) Day 8 hESC-ECP characteristics assessed against a target profile determined at the start of the study are shown; n = 21 replicates. (C) qPCR-detected expression of selected pluripotent (NANOG, OCT4, and SOX2) and endothelial (CD31, KDR, and CD34) genes in differentiated RC11 cells shows the downregulation of pluripotency and acquisition of endothelial phenotype in comparison to mRNA from human umbilical vein endothelial cells (HUVECs) as a positive control. Data are shown as 2ΔCt × 1,000 compared to the housekeeping gene β-actin. hESC data are n = 4 biological replicates assayed in triplicate, HUVEC n = 3 in triplicate; *p < 0.05, **p ≤ 0.01, and ***p ≤ 0.001 denote significance compared to d0; †p < 0.05, ††p ≤ 0.01, and †††p ≤ 0.001 denote level of significance compared to HUVECs using one-way ANOVA with Tukey’s post hoc test. All data represent mean ± SEM.

We first set a product profile requiring that greater than 25% of differentiated cells co-express the mature endothelial markers CD31/CD144, and less than 0.5% of the final product are double positive for pluripotent markers SSEA4/TRA1-81 or SSEA3/TRA1-60. As shown in Figure 1A and summarized in Figure 1B, assessment of 21 independent hESC-ECP preparations, generated using the protocol, reproducibly and robustly exceeded our requirements with 60.6% ± 3.0% CD31^+^/CD144^+^ cells whereas residual pluripotent cells were only detected in 3/21 samples, giving a mean of <0.01% for either combination of markers (SSEA4/TRA1-81 or SSEA3/TRA1-60). Of those 3 samples, the highest proportion of double-positive cells was 0.097% (SSEA3/TRA1-60); in all other cases (18/21), the percentage was lower than the isotype control. The hESC-ECPs were able to form tubules on Matrigel (Figure S1B), and this ability, in addition to CD31/CD144 expression, was not affected by 7 hr (during transportation) at room temperature on day 7 of the protocol (Figure S2). hESC-ECPs were subsequently used for *in vitro* and *in vivo* characterization.

**Figure 2:**
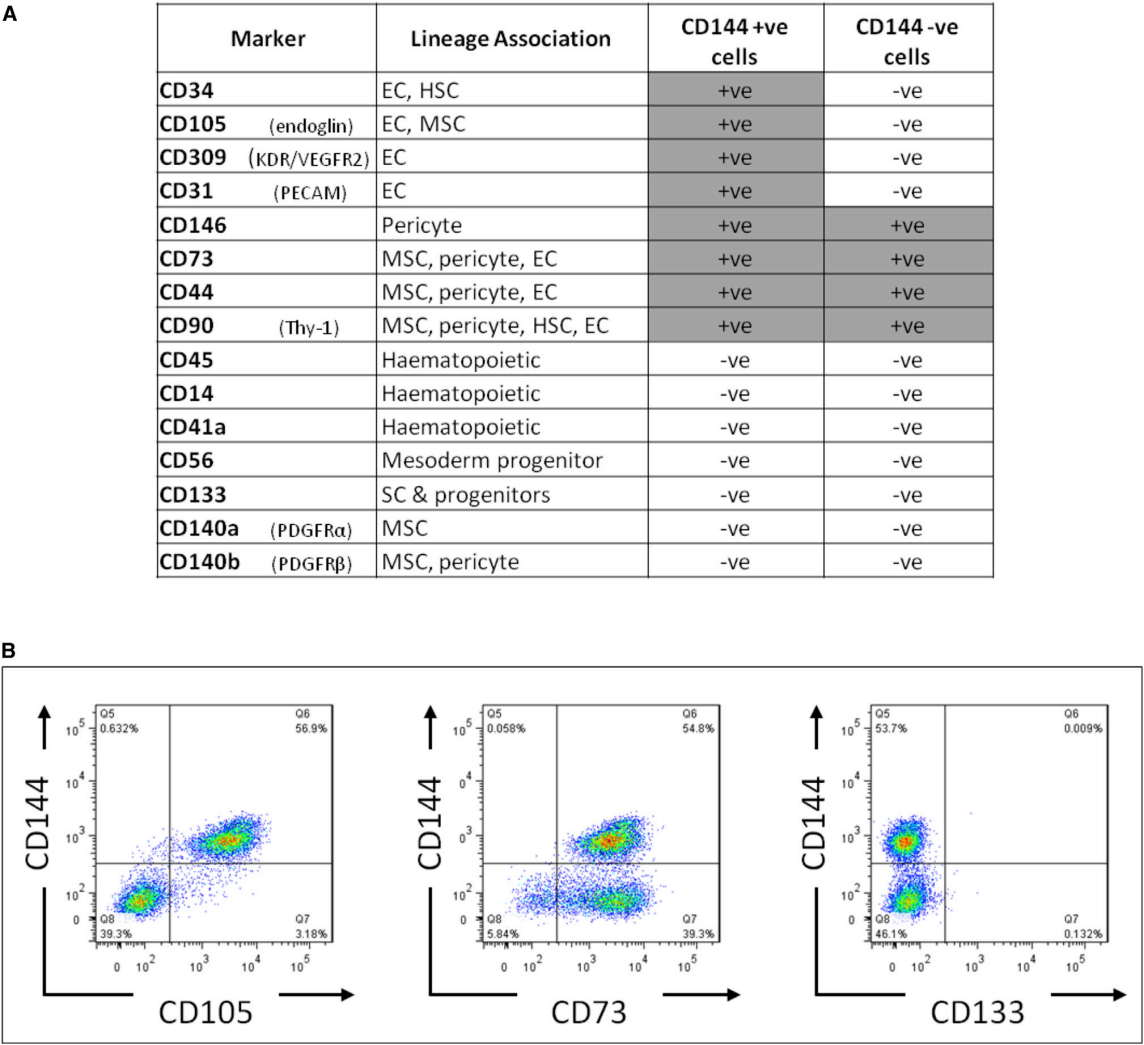
Extended Surface Marker Analysis of Differentiated RC11 hESC-ECP An extended panel of surface markers known to be expressed in mesodermal, mesenchymal, hematopoietic, or pericyte differentiation of hESC was also assessed on the CD144/CD31 population (CD144^+^) and on those cells not expressing CD144 (CD144^−^). (A) Summaries of the data from 3 biological replicates are shown. (B) Examples of additional markers demonstrating 3 different patterns of expression are shown. Left panel, only CD144^+^ cells are positive for another marker (CD105); middle panel, CD144^+^ and CD144^−^ cells are both positive for other marker (CD73); right panel, CD144^+^ and CD144^−^ cells are both negative for other marker (CD133). EC, endothelial cell; HSC, hematopoietic stem cell; MSC, mesenchymal stromal cell; SC, stem cell. All data represent mean ± SEM.

To determine the identity of the remaining 40% of cells that were not double positive for the characteristic endothelial combination of CD31/CD144, we assessed expression of a wider panel of surface markers by fluorescence-activated cell sorting (FACS), with a focus on mesenchyme, pericyte, and hematopoietic cell markers. On day 8 of differentiation, all cells positive for CD144 were also positive for CD31; therefore, we assessed combinations of CD144 and additional markers. All of the additional markers were expressed on either ≥95% or on ≤5% of cells, no bi-modal populations were observed, and, therefore, markers were scored as positive or negative (Figure 2A). The pattern of staining fell into 3 groups (Figure 2B): markers typically observed on less mature endothelial cells and co-expressed on only CD144-positive cells (e.g., CD34, CD105, and CD309); MSC and pericyte markers on all cells (e.g., CD73, CD44, CD90, and CD146); and hematopoietic/earlier progenitors that were negative on all cells (e.g., CD14, CD45, CD56, and CD133). Analysis of mRNA from the day 8 population also demonstrated downregulation of pluripotent-associated genes to similar levels to those of human umbilical vein endothelial cells (HUVECs). HUVECs were chosen as a control as they are fetal endothelial cells and therefore closer in terms of developmental age to hESC-ECPs than adult ECs. Expression profiles of endothelial genes also reflected the immature stage of the hESC-ECP; in hESC-ECP, CD31 and CD144 increased over time (8 days) to levels similar to those in HUVECs, whereas expression levels of KDR and CD34 increased to levels that were significantly higher than in HUVECs (Figures 1C and S1A). As the unmanipulated (non-purified cell product) produced by this protocol is the one intended for clinical use, the total heterogeneous cell population was used throughout this study and referred to as hESC-ECP due to the majority endothelial phenotype. Both the endothelial and non-endothelial (based on CD144 sorting) components of this heterogeneous population expressed genes associated with angiogenesis (Figure S10).

### Longitudinal Biodistribution of Transplanted hESC-ECPs

**Figure 3:**
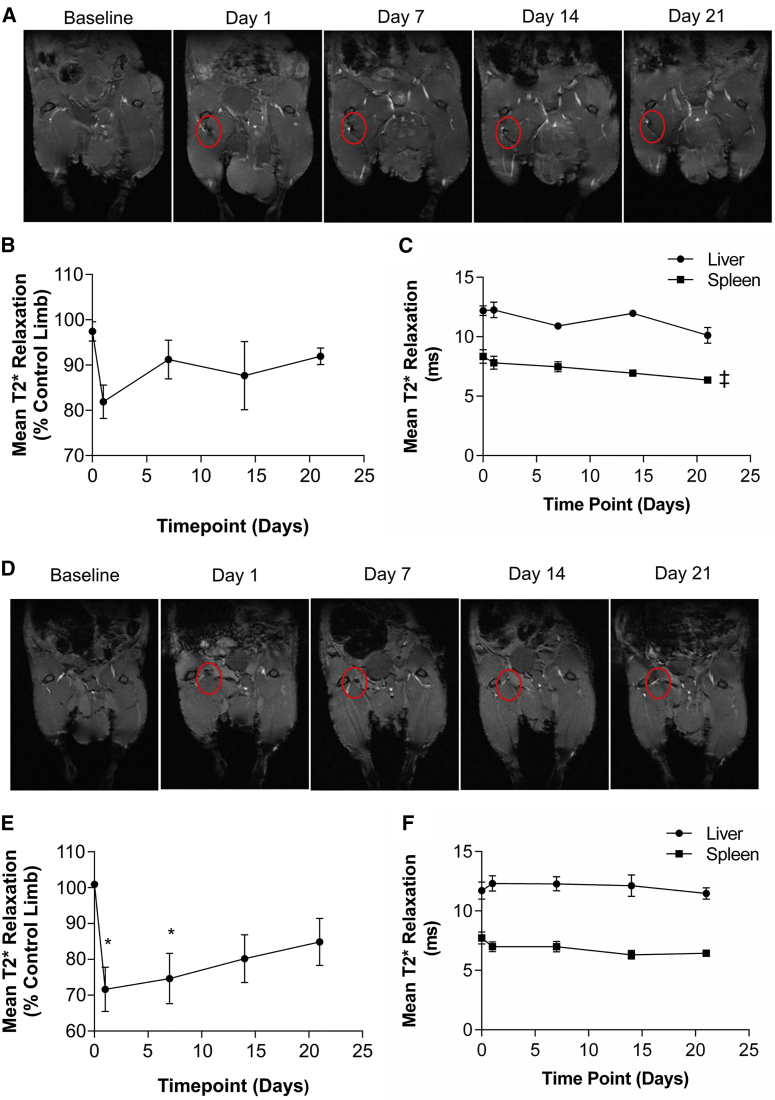
Longitudinal Tracking of hESC-ECP in Control and Ischemic Limbs (A) Representative images of control (without limb ischemia), nude (Crl:CD1-Foxn1^nu^) mouse limbs post-injection of SPIO-labeled hESC-ECP with dark regions indicating SPIO-mediated signal suppression (circled), taken from the 2^nd^ echo of a T2* mapping sequence. (B) Quantification of mean T2* relaxation time within the injection site relative to the contralateral control limb is shown; n = 7. (C) Quantification of mean T2* relaxation time within the liver and spleen is shown; n = 7; ‡p < 0.05 by one-way ANOVA paired for signal suppression over time. (D) Representative images of ischemic mouse limbs post-injection of SPIO-labeled hESC-ECP are shown, with dark regions indicating SPIO-mediated signal suppression (circled), taken from the 2^nd^ echo of a T2* mapping sequence. (E) Quantification of mean T2* relaxation time within the injection site relative to the contralateral control limb is shown; n = 7; *p < 0.05 versus baseline; by one-way ANOVA paired with post hoc Dunnett’s test. (F) Quantification of mean T2* relaxation time within the liver and spleen is shown; n = 7. All data represent mean ± SEM.

It was hypothesized that transplanted hESC-ECPs would be present and engraft for the duration of the study (day 21). Therefore, long-term distribution of hESC-ECP was assessed in the first instance using superparamagnetic iron oxide nanoparticle (SPIO) labeling of hESC-ECP in combination with MRI. hESC-ECP uptake of SPIO was optimized to ensure sufficient contrast without affecting hESC-ECP viability or function (Figure S3). Following injection, MRI imaging of labeled cells in mice without limb ischemia (LI) demonstrated a moderate signal suppression due to the presence of SPIO at the injection site on day 1, which returned to baseline values by day 7 (Figures 3A and 3B). This did not reach statistical significance by one-way ANOVA. The liver and spleen were also imaged as these organs are suitable for T2* mapping and are associated with uptake of cells from the circulation and SPIO clearance (Figure 3C). There was no signal suppression within the liver and a minor time-dependent suppression of signal within the spleen. hESC-ECP tracking in nude mice with LI (Figures 3D and 3E) demonstrated greater signal suppression (∼30% versus ∼20%), which was maintained for longer when compared with unoperated mice (Figure 3B). Significant signal suppression was evident at 1 and 7 days post-injection versus baseline, with no statistically significant differences at days 14 and 21 (Figure 3E). In these mice, there was no signal suppression within the liver or spleen (Figure 3F).

### Short-Term Biodistribution of Transplanted hESC-ECPs

**Figure 4:**
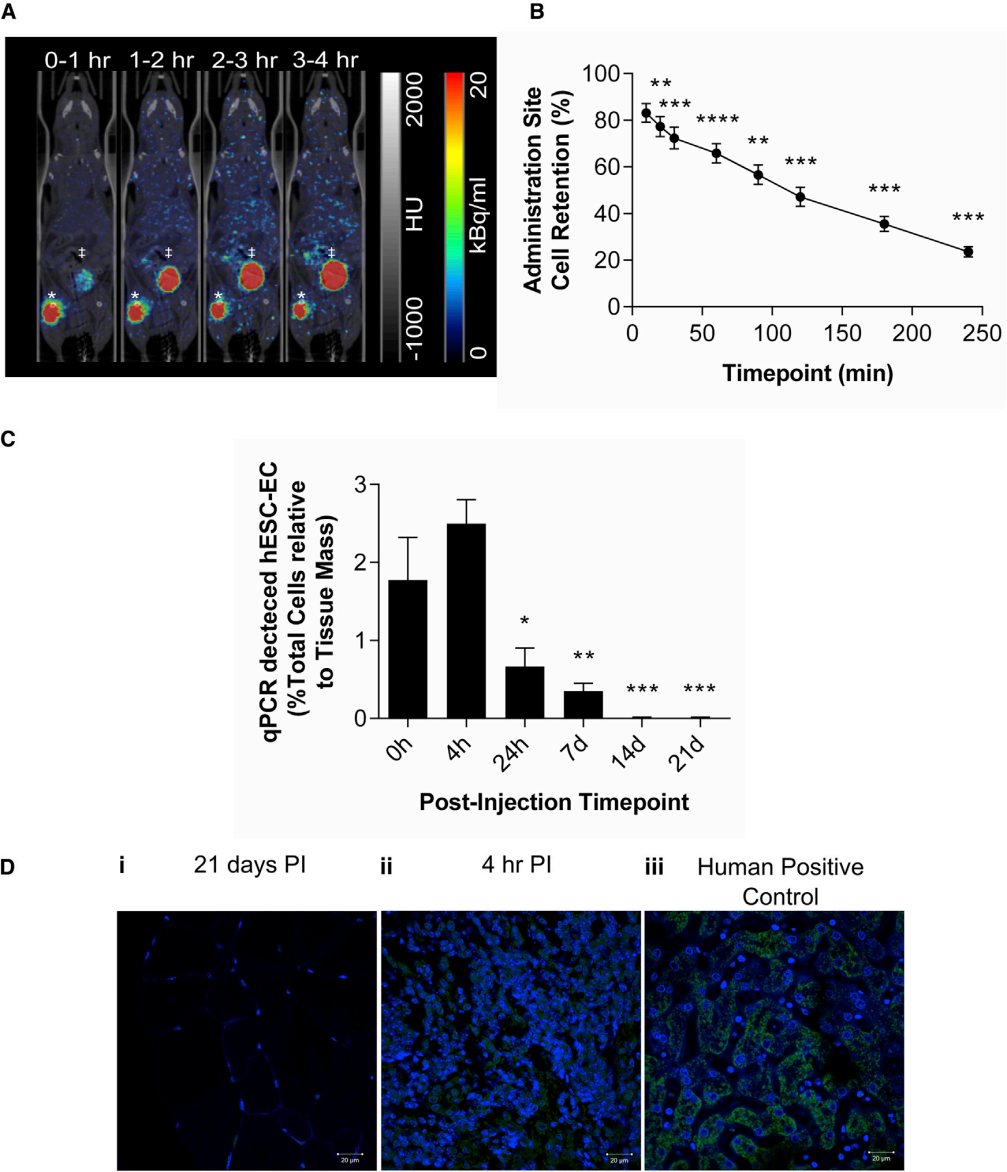
PET, qPCR, and Histological Detection of hESC-ECP within Ischemic Hindlimbs at Early and Late Time Points (A) Representative average time frames of nude (Crl:CD1-Foxn1^nu^) mice with hindlimb ischemia up to 4 hr post-injection. *Injection site; ‡urinary bladder. (B) Estimation of cell retention within the injection site is shown; n = 5; **p ≤ 0.01, ***p ≤ 0.001, and ****p ≤ 0.0001 versus baseline by one-way ANOVA paired with post hoc Dunnett’s test. (C) qPCR quantification of human DNA in ischemic hindlimbs harvested at time points corresponding to imaging studies is shown. n = 6; *p < 0.05, **p ≤ 0.01, and ***p ≤ 0.001 versus baseline by one-way ANOVA with post hoc Dunnett’s test. (D) Immunofluorescence for human mitochondria (green) within (i) ischemic muscle 21 days post-injection (PI), (ii) ischemic muscle 4 hr post-injection, and (iii) human positive control (liver) is shown. Nuclei counterstained with DAPI (blue) are shown; images representative of n = 3. All data represent mean ± SEM.

Because long-term tracking suggested that the hESC-ECP was lost from the hindlimb within the first week following injection, detailed, dynamic analysis of cell distribution immediately following injection was necessary. In order to track the initial distribution of hESC-ECP within ischemic hindlimbs of nude mice, cells were labeled with ^18^F-FLT and imaged by dynamic positron emission tomography (PET). We previously determined optimal hESC-ECP labeling with ^18^F-FLT^19^ and showed that this did not affect hESC-ECP viability, proliferation, or function (Figure S4). Dynamic PET imaging post-injection of labeled hESC-ECP detected signal in the mouse hindlimb that dropped rapidly over the 4 hr scanning period (10, 20, 30, 60, 90, 120, 180, and 240 min; Figures 4A and 4B). This indicated a significant loss of transplanted cells from the injection site, with 23.5% ± 2.2% cell retention at 4 hr. These data were corrected for radioactive decay as well as leakage of free radiotracer from the cells (which accumulated within the elimination organs; Figure S5A). The maximum accumulation of signal within the other measured sites was 0.28%ID, 99-fold lower than in the elimination organs at 4 hr (Figure S5B).

### qPCR and Histological Detection of Human Cells

To validate the cell distribution profiles demonstrated using MRI and PET, nude mice with LI received an intramuscular injection of hESC-ECP and were sacrificed at time points corresponding to the imaging studies. The percentage of human cells within each ischemic hindlimb was calculated at each time point (Figure 4C), using a standard curve with decreasing human:mouse DNA ratios (Figure S6). There was no decrease in the presence of human cells within the first 4 hr post-injection and at 24 hr and 7d hESC-ECP was still present within the ischemic hindlimb, even if at a reduced amount (0.7% ± 0.2% [24 hr] and 0.4% ± 0.1% [7d] versus 1.8% ± 0.6% at 0 hr; p < 0.05). At days 14 and 21, human cells were no longer present within the ischemic hindlimb. Qualitative analysis of human-specific mitochondrial staining within ischemic muscle harvested at 21 days post-injection demonstrated no significant signal compared to positively stained, cell-dense patches of hESC-ECP in tissue harvested 4 hr post-injection and human control tissue (Figure 4D).

### Intramuscular Injection of hESC-ECP Improves Post-ischemic Blood Flow Recovery in Immunocompromised and Immunocompetent Mice

**Figure 5:**
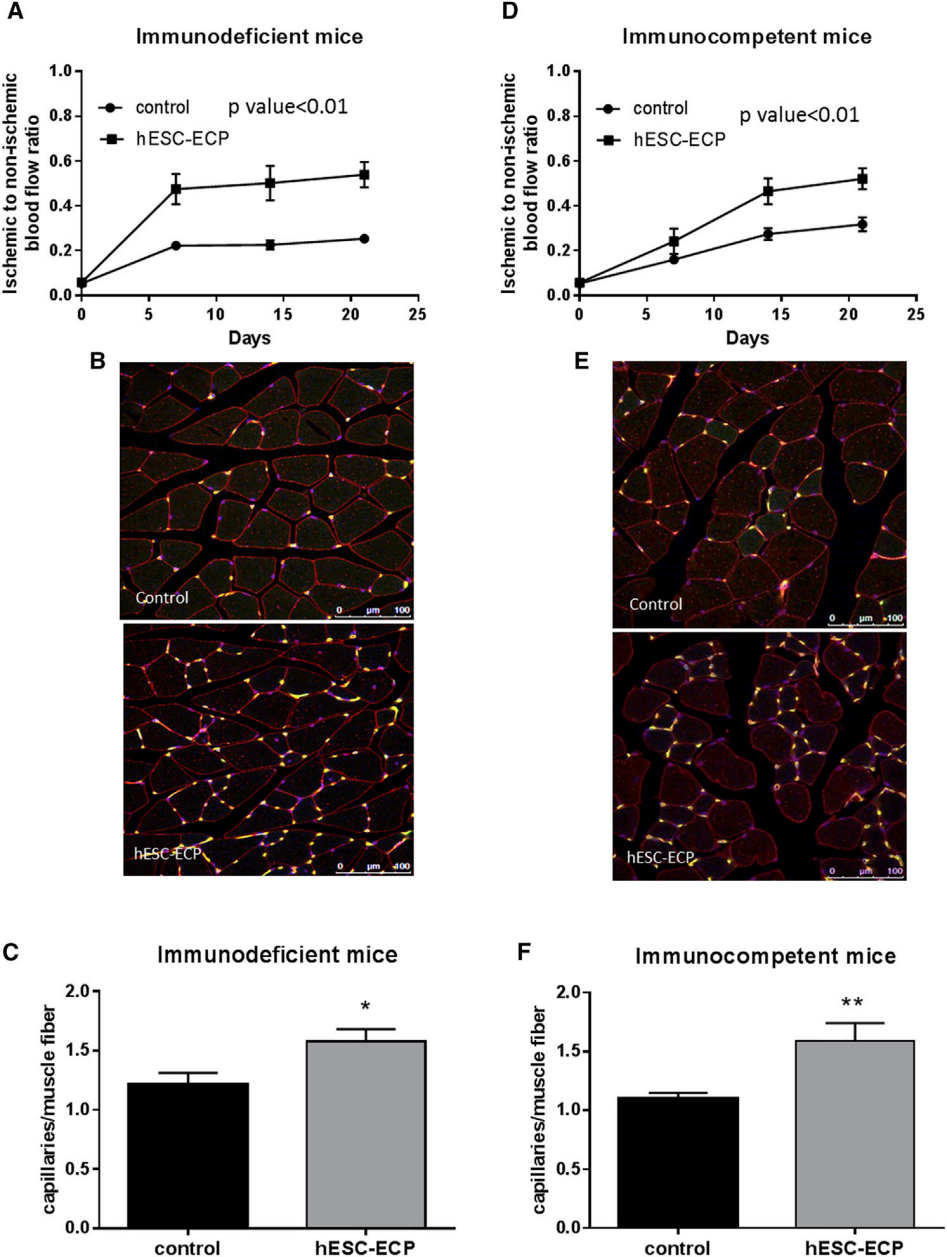
hESC-ECP Significantly Increases Post-ischemic Blood Flow Recovery and Capillary Density in Nude Crl:CD1-Foxn1^nu^ and Immunocompetent CD1 Mouse Model of Hindlimb Ischemia Foot perfusion was assessed following hindlimb ischemia in immunodeficient (Crl:CD1-Foxn1^nu^) and immunocompetent mice treated with vehicle (EBM-2, control) or hESC-ECP. The rate of blood flow recovery was expressed as the ratio of ischemic to contralateral limb blood flow at 0, 7, 14, and 21 days post-treatment for (A) immunodeficient, p < 0.01, n = 12, correlated outcome analysis and (D) immunocompetent mice, p < 0.01, n = 21, correlated outcome analysis. Representative images demonstrating endothelial cell (green), muscle (wheat germ agglutinin; red), and nuclear (DAPI; blue) markers in control (top) and hESC-ECP-treated (bottom) adductor muscles from (B) immunodeficient and (E) immunocompetent mice are shown. Scale bar indicates 100 μm. Capillary density expressed as the ratio of capillaries/muscle fiber in (C) immunodeficient, n = 7, p < 0.05, Mann-Whitney U test, and (F) immunocompetent mice, n = 12, p < 0.05, Mann-Whitney U test, is shown. All data represent mean ± SEM.

The therapeutic efficacy of hESC-ECP was tested in different mouse models of acute hindlimb ischemia. We first investigated the efficacy of hESC-ECP in immunocompromised Crl:CD1-Foxn1^nu^ mice, which are particularly indicated for xenotransplantation. Laser Doppler measurement showed reduction of blood flow in ischemic limb shortly after surgical occlusion of femoral artery, confirming successful induction of acute LI. Follow-up showed progressive recovery of blood flow with significantly higher post-ischemic hemodynamic recovery in hESC-ECP-treated mice (Figures 5A and S7A). Furthermore, cell-treated mice showed increased capillary density within ischemic limb muscles 21 days post-surgery, as demonstrated by immunofluorescent staining (Figures 5B, 5C, and S7B).

To determine whether hESC-ECP therapy requires an immunocompromised host, the same protocol was repeated in immunocompetent CD1 mice with hindlimb ischemia. hESC-ECP administration induced a significant improvement in hindlimb blood flow when compared to control CD1 mice (Figures 5D and S7C). There was also a significant increase in capillary density within adductor muscles of hESC-ECP-treated mice compared to controls (Figures 5E, 5F, and S7D).

### hESC-ECP Improves Post-ischemic Blood Flow Recovery in Diabetic Mouse Models of Hindlimb Ischemia

**Figure 6:**
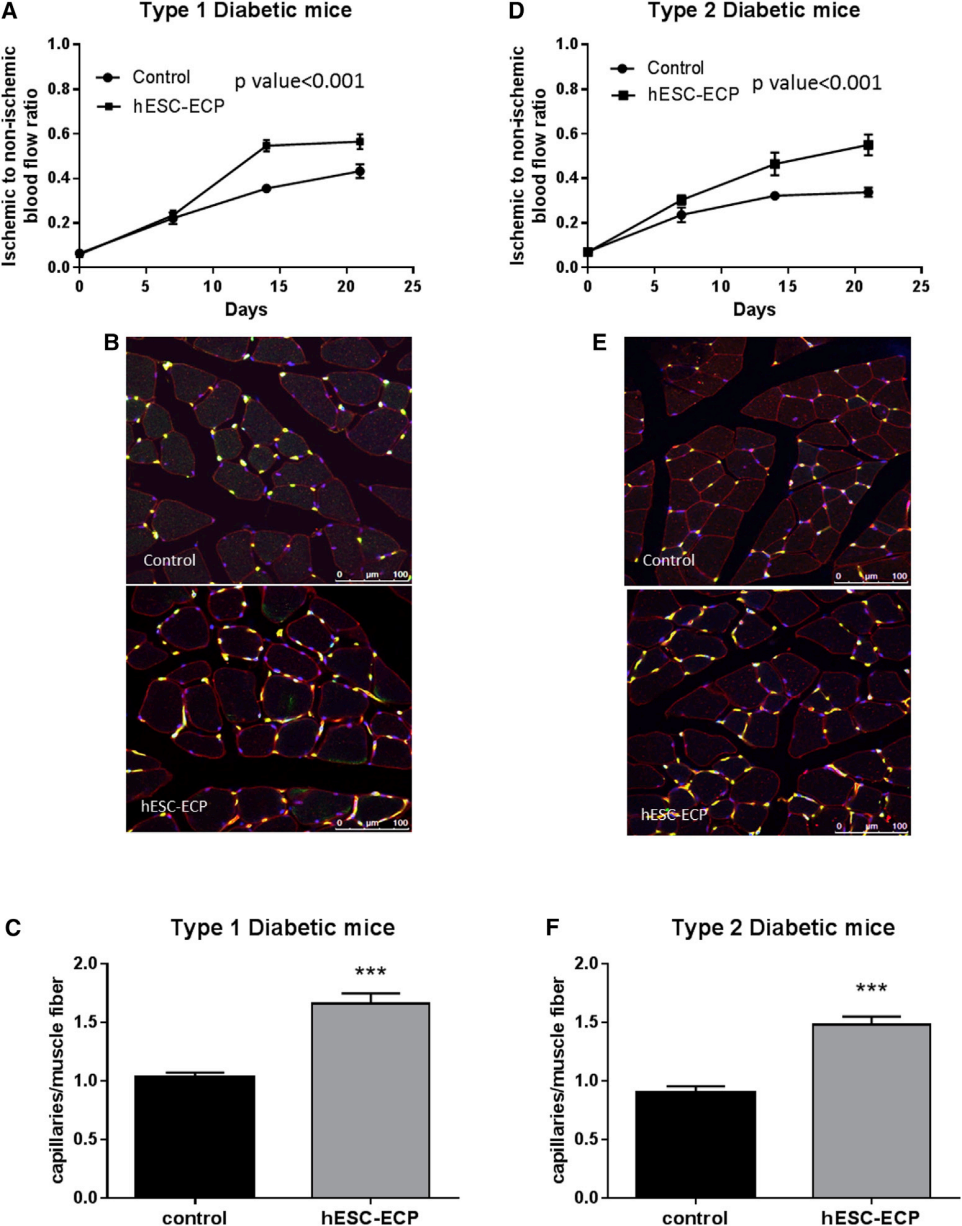
hESC-ECP Significantly Increase Post-ischemic Blood Flow Recovery in Type 1 and Type 2 Diabetic Mice Foot perfusion was assessed following hindlimb ischemia in streptozotocin (STZ)-induced type 1 diabetic CD1 mice and in BKS.Cg-Dock7m+/+LeprdbJ (db/db) type 2 diabetic mice treated with either vehicle (EBM-2; control) or hESC-ECP. Blood flow recovery was expressed as the ratio of ischemic to contralateral blood flow at 0, 7, 14, and 21 days post-treatment for (A) STZ-induced type 1 diabetic mice and (D) db/db type 2 diabetic mice, n = 12, p < 0.001, correlated outcome analysis. Representative images demonstrating endothelial cell (green), muscle (wheat germ agglutinin; red), and nuclear (DAPI; blue) markers in control (top) and hESC-ECP-treated (bottom) adductor muscles from (B) STZ-induced type 1 diabetic mice and (E) db/db type 2 diabetic mice are shown. Scale bar indicates 100 μm. (C) Capillary density expressed as the ratio of capillaries/muscle fiber in STZ-induced type 1 diabetic mice, n = 7, p < 0.001, and (F) db/db type 2 diabetic mice, n = 8, p < 0.001, Mann Whitney U test, is shown. All data represent mean ± SEM.

As the majority of patients with CLI have diabetes mellitus, which further impairs angiogenesis,^20^, ^21^ we tested the efficacy of hESC-ECP in type 1 and type 2 diabetic mouse models. We used streptozotocin-induced (STZ) type 1 diabetic CD1 mice and genetically modified db/db mice (BKS.Cg-Dock7m+/+LeprdbJ) with type 2 diabetes. Laser Doppler assessment of blood flow showed progressive recovery of blood flow in ischemic hindlimbs injected with hESC-ECP both in STZ type 1 diabetic mice (Figures 6A and S8A) and in db/db type 2 diabetic mice (Figures 6D and S8C) compared with EBM-2-treated controls. Moreover, hESC-ECP increased capillary to myofiber number ratio both in STZ mice (Figures 6B and 6C) and in db/db mice (Figures 6E and 6F) compared with controls. hESC-ECP increased capillary density per mm^2^ in STZ mice only (Figures S8B and S8D).

### Comparison of hESC-ECP Efficacy Administered in CD1 Mice 3 Days after Establishment of Ischemia

**Figure 7:**
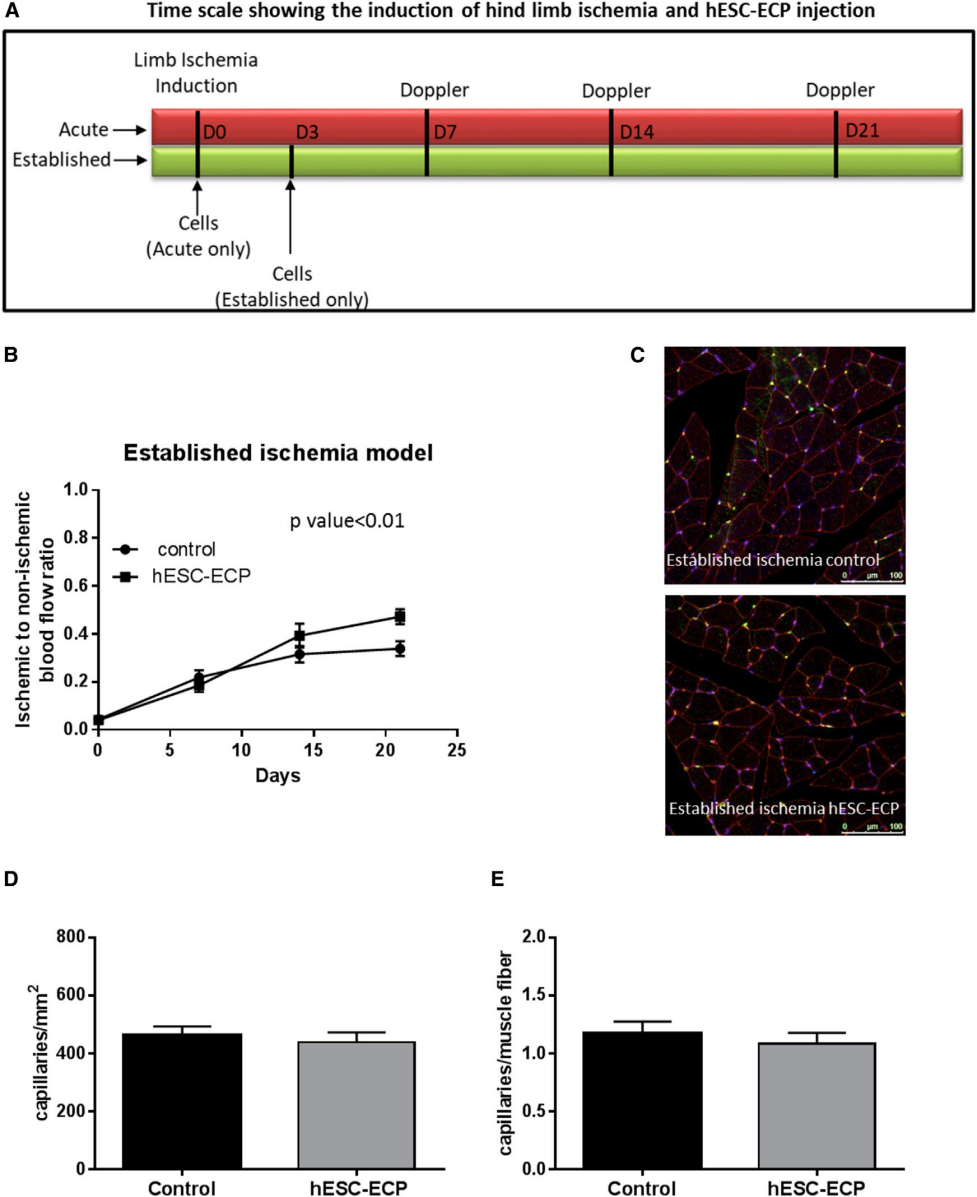
hESC-ECP Efficacy in CD1 Mice with Established Hindlimb Ischemia (A) Timescale of induction of hindlimb ischemia and cell therapy in acute and established ischemia models. (B) Blood flow recovery expressed as the ratio of ischemic to contralateral limb blood flow at 0, 7, 14, and 21 days after surgery in an established ischemia model, n = 9, p < 0.01, correlated outcome analysis, is shown. (C) Representative images demonstrating endothelial cell (green), muscle (wheat germ agglutinin; red), and nuclear (DAPI; blue) markers in adductor muscles are shown. Scale bar indicates 100 μm. (D) Capillary density expressed as the ratio of capillaries/ mm^2^ and (E) capillaries/muscle fiber in established ischemia, n = 5, p = NS for any comparison, Mann Whitney U test, is shown. All data represent mean ± SEM.

To get closer to the clinical scenario, in which cell therapies for CLI involve cell transplant into established ischemic conditions, we investigated whether hESC-ECPs were still effective when delivered at 3 days post-ischemia induction (Figure 7A). CD1 mice that received hESC-ECP on day 3 post-ischemia induction showed increased blood flow recovery (Figures 7B and S9). However, hESC-ECP failed to improve capillary density at day 21 from surgery (Figures 7C–7E).

## Discussion

For the first time, we have developed a GMP-compatible protocol using clinical-grade cells and have shown that this cell product consistently stimulates increased recovery of blood perfusion to the ischemic limb in a number of complementary animal models of limb ischemia, including in animals with diabetes. Cell tracking experiments demonstrated clearance of transplanted hESC-ECP within 2 weeks post-injection, suggesting a paracrine mechanism of action. Combined, this study paves the way for translation of this method into the clinic.

Through the adaptation of previously published methodology,^14^ we have developed a GMP-compatible protocol using a quality management system that is free from xenobiotic reagents, using methods that can be simply completed in a clean room environment and importantly utilizing a clinical-grade hESC line (RC11). Generally, hESC-EC differentiation protocols have a low efficiency, most usually between 10% and 30%,^22^, ^23^, ^24^, ^25^ resulting in the need for the final cell product to be sorted using immunomagnetic beads, which introduces additional regulatory and cost considerations. The highly efficient system reported by Patsch et al.^14^ relies on a sorting step that generates 80% PSC-ECs. Our newly adapted protocol presented in this study results in 60% CD31^+^/CD144^+^ cells with <0.05% pluripotent markers; thus, this improvement in endothelial-marker-positive cells removes the need for cell sorting, improving the translatability of this cell product. In addition, during this multicenter study, hESC-ECPs were successfully transported by couriers for ∼7 hr and ∼370 miles (from Edinburgh to Bristol) without impairing their function, which is an important consideration for the translation of this cell product. Our differentiation method produced a preparation of endothelial progenitors, in addition to a smaller population of cells that are not overtly endothelial, expressing markers characteristic of mesenchymal stromal cells (MSCs) and pericyte lineages. Pericytes perform a supporting role during angiogenesis and have been shown to improve reperfusion in murine hindlimb ischemia previously.^26^ Therefore, it is possible that both the EC progenitor population and those expressing MSC and pericyte characteristics may contribute to the efficacy of this product, although this study has not assessed this aspect. Overall, the important point to note is that this cell product has shown robust efficacy, and it is intended to be used as a heterogeneous population for clinical translation. In addition, this is the first study to describe a fully GMP-compatible methodology using clinical-grade cells, which has a high endothelial differentiation efficiency and the ability to withstand the logistics required as part of a multicenter project.

The next component of this study was to assess the retention and distribution of transplanted hESC-ECP using clinically relevant imaging techniques, the first of which was the use of SPIO labeling and MRI for longitudinal tracking. For consistency with previous intervention studies, the cell product was administered using the standard intramuscular cell delivery method (i.e., along and/or near the projection of the adductor muscle^8^, ^11^, ^12^, ^17^, ^26^, ^27^, ^28^, ^29^) previously shown to increase blood flow in the preclinical model of limb ischemia.^8^, ^11^, ^12^, ^17^, ^26^, ^28^, ^29^ There is molecular evidence of ischemia in the adductor in this model, although it is likely that studies in patients will require cell injections at numerous sites.^16^ First, we demonstrated that retention of cells within ischemic muscles, in which the SPIO signal was present up to 7 days post-injection, was enhanced in comparison with healthy skeletal muscle, which is encouraging for use of cells in the clinical setting. However, at 14 days, labeled cells were no longer detectable despite their ability to improve reperfusion and capillary density. Therefore, with MRI suggesting a fast clearance of hESC-ECP, it was evident that the early tracking of transplanted hESC-ECP was vital. Short-term cell tracking was developed using dynamic PET imaging of ^18^F-FLT-labeled hESC-ECP. In agreement with our MRI findings, PET demonstrated a rapid clearance of signal over the first few hours. In addition, qPCR and histology directly confirmed this early clearance profile, with the cells no longer detectable by the 14-day time point. These results suggest the prevalence of paracrine mechanisms behind hESC-ECP induced improvement in foot reperfusion. Previous and independent preclinical cell therapy studies^28^, ^30^ already demonstrate improved perfusion of blood to the ischemic hindlimb in the absence of direct engraftment of transplanted cells. However, it should be noted that, in other instances within the heart, extensive engraftment has been reported after hESC-CM transplantation.^13^, ^31^ A strategy to improve the post-transplant survival and retention of our GMP-compatible cells in the future could utilize biological matrix or scaffolds, such as the recently published extracellular-matrix-mimicking peptide amphiphile nanomatrix gel.^17^

To translate hESC-ECP into the clinic, it is imperative to robustly demonstrate their efficacy in a number of models. To our knowledge, this is the first preclinical study to demonstrate the efficacy of hESC-ECP in several clinically relevant models (including type 1 and type 2 diabetes and cell transplant in muscles with established ischemia) in addition to undertaking detailed cell biodistribution studies. Moreover, another important question this study approached was whether transplanted hESC-ECP could improve ischemic limb reperfusion in the presence and absence of an intact immune response. In terms of translation, it is envisaged that allogenic hESC-ECP therapy will take place alongside transient immunosuppression of the patient.^32^ However, in this study, we demonstrate that hESC-ECP can improve reperfusion to the ischemic foot and increase the capillary density within the skeletal muscle in both immunocompromised (nude) and immunocompetent (CD1) mice. Demonstration of efficacy in the presence of an intact immune response is encouraging and suggests that it may be possible to deliver these cells in the absence of immunosuppression in the future. Notably, other clinical trials, such as the PISCES trial, deliver neural stem cells into the brain in the absence of immunosuppression and have reported no immunological or cell-related adverse events.^33^ The next question to be approached by this study was whether hESC-ECP could elicit their beneficial effects when administered during established ischemia, as opposed to administration in the period immediately after the LI procedure. This experimental design is currently lacking in many preclinical studies,^8^, ^9^, ^10^, ^11^, ^12^ in spite of the fact that, in clinical trials and practice, hESC-ECP will be transplanted into muscles that are chronically ischemic. hESC-ECP administered on day 3 significantly increased reperfusion to the foot by day 21. However, unlike the other LI experiments, no significant improvement was seen in capillary density. This is perhaps not surprising, as arteriogenesis and angiogenesis are distinct processes in this model,^34^ both of which are important to vascular regeneration.^34^ In addition, we have recently published findings using this model to compare the recovery response in various genotypes, which demonstrated that reperfusion of blood to the foot does not directly correlate with changes in capillary density in this model.^35^ Therefore, evaluation of both capillary density and foot perfusion is important in the preclinical assessment of cell therapies, although perhaps greater emphasis should be placed on the latter.

hESC-ECP therapy is also likely to be undertaken in the presence of diabetes mellitus. Diabetes-induced impairment of angiogenesis has long been known,^20^, ^21^ and therefore, we assessed the impact of this condition on the efficacy of hESC-ECP. In both type I and II diabetes mellitus, hESC-ECP significantly improved foot reperfusion and skeletal muscle capillary density. Therefore, this study demonstrates that our GMP-compatible hESC-ECP can rescue blood flow within the ischemic limb and improve capillary density in a number of clinically relevant models, suggesting that these cells may prove useful in humans, warranting further study in the clinical setting.

Whereas there are some cell therapy products, such as PLX PAD (ClinicalTrials.gov identifier: NCT03006770), under investigation in phase III clinical trials, there remains no US Food and Drug Administration (FDA)-approved gene or cell therapy for the treatment of CLI. This may be, in part, due to the fact that these therapies must demonstrate an increase in amputation-free survival rather than other benefits, such as reduction in pain and ulcer healing, in order to be approved.^36^ In two recently published retrospective meta-analyses,^7^, ^37^ cell therapy studies produced no overall reduction in limb amputations. The only significant improvement demonstrated was a decrease in pain score following cell treatment versus placebo.^37^ The failure of these studies could be attributed to a number of factors, including an overambitious and imprecise primary endpoint, undefined populations, differing production methods, and/or differing administration regimens (route, dose, and timing). Increasingly, clinical trials are moving away from intra-arterial and intravenous administration toward intramuscular injections across as many as 30 sites.^38^ These refinements are crucial to the progression of the field. Also, we believe the use of a robust, well-defined pluripotent cell-derived product may result in more favorable results, and clearly our study warrants such translation using a well-defined population of cells. To date, trials of hESC-derived products are underway in several conditions, including macular degeneration, type I diabetes mellitus (for re-establishing insulin-secreting beta cells), spinal cord injury, and heart failure,^39^ with the limited results published so far demonstrating their safety.^40^ Clinical assessment of hESC-ECP in CLI will be an important step in the field and may offer renewed hope for therapeutic angiogenesis. This approach may also prove applicable to the myocardium post-myocardial infarction.^41^

Whereas the utility of hESC-ECP and pluripotent cell-derived products offers obvious advantages over multipotent or somatic cells, they are faced with disadvantages, such as the possible requirement for immunosuppression and risk of teratoma formation.^5^ One limitation of our study is that we are yet to undertake assessment of potential teratoma formation. Whereas we have demonstrated that cells expressing pluripotent markers are rare in our final hESC-ECP, teratoma or tumor formation assessment is planned before clinical translation. In addition, all preclinical studies are limited by their relation to the clinical scenario. However, where possible, we have included the most relevant models in our study. Future studies using larger animal models will allow for more clinically relevant outcome measures to be assessed, such as ankle brachial index and transcutaneous oxygen pressure,^42^, ^43^ as well as allowing for further optimization of administration route and dose. It is unlikely that future clinical trials will inject cells solely into ischemic or non-ischemic areas. Areas with intermediate levels of ischemia (between healthy and necrotic) are likely to present better options for cell delivery. Given the difficulties in targeting such areas in the ischemic mouse hindlimb, we have limited ourselves to essential proof-of-concept studies. It is anticipated that future clinical safety and feasibility studies will address optimum route of administration, possibly in patients with no other treatment options available. Finally, our differentiation protocol has been validated with other lines, including H9 hESC and an iPSC line NAS2^44^ (Figures S11 and S12) with similar results. This differentiation is robust and reproducible, and we are now using it as a model in which to study the role of non-coding RNAs during endothelial commitment and differentiation. Ultimately though, hESC-ECP derived from RC11 cells will be the product that we intend to assess in a first-in-man trial; therefore, we have concentrated our efficacy and cell-tracking efforts on that clinical-grade hESC line. We are now considering scale-up in a closed culture system, such as CellStack or Hyperflask (Corning, USA), and cryopreservation of the product to ease manufacturing and distribution schedules.

### Conclusions

In this study, we present a GMP-compatible hESC-ECP production protocol, with precise characterization of the cellular subsets, which we aim to use as a heterogeneous endothelial cell product. Positive efficacy studies in clinically relevant models of limb ischemia demonstrate the robust efficacy of our product, with the addition of detailed cell-tracking studies pointing toward a paracrine mechanism of action. Efforts should now be focused toward a first-in-man clinical trial utilizing the results from this study.

## Materials and Methods

### Cell Culture and Endothelial Differentiation

A clinical -grade hESC line, RC11 was used throughout and was maintained in StemPro complete medium (Life Technologies, UK) with 20 ng/mL basic fibroblast growth factor (bFGF; R&D Systems, USA). Cultures were passaged when confluent (every 6 or 7 days) using EZPassage disposable stem cell passaging tools. Karyotype was regularly checked and by Giemsa (G)-band analysis was found to be normal in all 30 cells examined, excluding more than 10% mosaicism at 95% confidence (Cytogenetics Laboratory, UK).

Endothelial differentiation was based on modifications to the method by Patsch et al.^14^ A detailed method and reagent list can be found in Supplemental Information. Briefly, on day 0 one T25cm^2^ flask was pre-coated with recombinant human fibronectin (3 μg/cm^2^; R&D Systems, USA). RC11 at 80%–100% confluence were harvested using StemPro Accutase (Life Technologies, UK) and plated onto the coated surface at between 2 and 4 × 10^4^ cells/cm^2^ in mTeSR (Stem Cell Technologies, UK) with 10 μM Y27632 Rho-kinase inhibitor (Tocris, UK) before overnight incubation.

On day 1, the medium was removed and replaced with N2B27 medium (comprising 250 mL DMEM/F12/GlutaMAX medium + 250 mL CTS Neurobasal medium + 2.5 mL GlutaMAX [100×] + 10 mL CTS B27 + 5 mL CTS N2 + 0.5 mL β-mercaptoethanol) and sterile filtered (all from Life Technologies, UK) supplemented with 7 μM CHIR-99021 (GSK3β inhibitor; Sigma Aldrich, UK) and 25 ng/mL rhBMP4 (R&D Systems, USA). Cultures were left until day 4 with no further media change. On day 4, medium was removed and replaced with StemPro-34 SFM (Life Technologies, UK) supplemented with 200 ng/mL vascular endothelial growth factor (VEGF)-165 (R&D Systems, USA) and 2 μM forskolin (Sigma Aldrich, UK). Medium was changed daily to day 6. On day 6, medium was removed and adherent cell layers were washed with DPBS and detached using TrypleExpress (Life Technologies, UK). Washed cells were replated into fresh, T25, or T75 flasks with no fibronectin or other matrix at 4 × 10^4^ cells/cm^2^ in modified EGM-2 medium (EBM-2 + EGM-2 SingleQuots [Lonza, Switzerland] omitting the FBS and VEGF) supplemented with 1% vol:vol human AB serum (Sigma-Aldrich, UK or SNBTS) and 50 ng/mL VEGF-165. Medium was removed and replaced on day 7. On day 8, spent medium was removed and cells were harvested with Tryple Express for *in vitro* analysis. If cells were to be used for *in vivo* assays, they were not harvested, but flasks were completely filled with modified EGM-2 without the addition of VEGF and AB serum for expedited transport on day 7. Cells were transported in T75 flasks packaged in polystyrene boxes at ambient temperature by dedicated courier to ensure minimal disruption. On arrival, medium was removed and replaced with complete modified EGM-2, including AB serum and VEGF, and flasks incubated overnight for use on day 8. Cells were assessed for cell number and viability before use.

### Cell Characterization

Cells were analyzed on d0 and d8 using flow cytometry, with the antibodies shown in Supplemental Information, using the BD Canto II (Becton Dickinson, USA). Data were analyzed with FlowJo software (FlowJo, USA). Cell counts and viability were performed using the Cell Countess I system (Life Technologies, UK). Matrigel assays were performed on day 8 cells^45^ with 1% AB serum and 50 ng/mL VEGF-165 included in the substrate and assessed after 3 and 6 hr. Expression of selected pluripotent and endothelial genes was assessed by real-time PCR on days 0, 6, and 8 of the differentiation protocol using the Taqman 7900 (Life Technologies, UK).

### Mouse Models of Unilateral Hindlimb Ischemia

All animal experiments were performed in accordance with the Animals (Scientific Procedures) Act (UK) 1986 and under the auspices of UK Home Office project and personal licenses held within University of Bristol/University of Edinburgh facilities.

Surgical procedures were performed under inhaled general anesthesia (isoflurane) and with appropriate peri-operative analgesic cover (buprenorphine). Unilateral LI was surgically induced by left femoral artery occlusion as previously reported.^46^ This procedure consists of ligation (6-0 silk suture) at two points and electrocoagulation of the upper part of the left femoral artery, leaving the femoral vein and nerve untouched. Mice were maintained for up to 21 days after surgery.

### Cell Administration

hESC-ECP was injected (1 × 10^6^ cells in three injection sites) into the ischemic adductor muscle. For the majority of protocols, cells were delivered in the ischemic muscle immediately after LI induction (acute treatment model). Engraftment studies were performed in mice with and without LI (*vide infra*). To get closer to the clinical scenario in which patients are mainly suffering from chronic ischemia, part of the efficacy studies were performed in subgroups of CD1 mice, which were subjected to cell therapy at 3 days post-ischemia induction (established ischemia model; *vide infra* and Figure 7A). In all aforementioned studies, control groups received the vehicle, EBM-2, rather than cells.

### Post-injection hESC-ECP Engraftment Analyses

To investigate the engraftment of hESC-ECP after intramuscular delivery, we combined *in vivo* short-term dynamic and longitudinal MRI imaging with qPCR to detect human cells and analyses of anti-human mitochondria immunofluorescence in the mouse muscles. Immunocompromised Crl:CD1-Foxn1^nu^ mice (aged 7 or 8 weeks at the time of LI operation; Charles River) were employed in these experiments.

#### Longitudinal MRI Tracking of hESC-ECP

MRI was used to assess the long-term (1–21 days) fate of SPIO-labeled hESC-ECP after injection in murine limb muscles. The protocol was developed in mice that had either not undergone surgery or that had undergone unilateral LI (aforementioned acute ischemia model). Optimal cell labeling conditions were investigated based on previously reported methods.^47^, ^48^ Briefly, 5 μg/mL protamine sulfate (Sigma-Aldrich, UK) was added to 2.5 μg Fe/mL of SPIO (FeraTrack, Miltenyi Biotec, Germany) and left to form a complex (15 min; room temperature). The SPIO-protamine complex solution (10 mL) was added to a T75 culture flask (Corning, USA) containing approximately 4 × 10^6^ on day 7 hESC-ECP and incubated (16 hr). The flask containing the SPIO-labeled hESC-ECP was then washed twice with DPBS (Lonza, Switzerland) before the cells were harvested using TrypleExpress (Life Technologies, UK). Cell density was then quantified by hemocytometer and adjusted to 1 × 10^6^ hESC-ECP/15 μL.

SPIO-labeled hESC-ECP were injected into the adductor muscle at three sites (total = 1 × 10^6^ cells in EBM-2). MRI scanning was performed 1, 7, 14, and 21 days after surgery. Mice were anesthetized with 1.8% isoflurane in oxygen/air (50/50; 1 L/min) and placed in a cradle (Rapid Biomedical, Rimpar, Germany), with their temperature maintained at 37°C throughout. All MRI experiments were performed using a 7-T horizontal bore nuclear magnetic resonance (NMR) spectrometer (Agilent Technologies, Yarnton, UK); specific imaging parameters are detailed in the Supplemental Materials and Methods. T2* maps were blinded before analysis using in-house software (MAPPED V3.4, University of Edinburgh), also described in the Supplemental Materials and Methods.

#### Short-Term Dynamic PET Cell Tracking

Dynamic PET allows sensitive tracking of ^18^F-FLT-labeled hESC-ECP over shorter timescales (≤4 hr). ^18^F-FLT was prepared using a standard FASTlab FLT Cassette (GE Healthcare, UK) and was formulated in 9% ethanol in water. Radiochemical purity was >99% for ^18^F-FLT. ^18^F-FLT-hESC-ECP labeling was carried out as previously described.^19^ Labeled hESC-ECPs were then harvested and suspended in EBM-2 at 1 × 10^6^ cells/15 μL for immediate administration.

^18^F-FLT-labeled hESC-ECP (1 × 10^6^ cells) were injected into the adductor muscle at three sites (total = 100–200 kBq/1 × 10^6^ cells), after which the surgical wound was closed using interrupted sutures (4/0 silk) and the animal was placed into the nanoPET/computed tomography (CT) scanner. Anesthesia was maintained throughout the imaging session using isoflurane (1.5%; 0.5:0.5 oxygen/nitrous oxide; 1 L/min). All PET data were acquired using a nanoPET/CT scanner (Mediso, Hungary). Post-administration of radiolabeled cells, a 240-min whole-body emission scan was obtained followed by a CT scan. Acquisition, reconstruction, and analysis were performed as previously described to calculate percentage cell retention.^19^

#### qPCR Detection of hESC-ECP in Mouse LI

Mice received unlabeled hESC-ECP (1 × 10^6^) at 3 injection sites in the adductor muscle immediately after LI and were culled 0 hr, 4 hr, 24 hr, 7 days, 14 days, or 21 days following surgery. Whole animal carcasses were snap frozen on dry ice prior to isolation of the entire hindlimb musculature to limit loss of transplanted hESC-ECP at the early time points. Entire hindlimb muscle tissue was homogenized and the DNA extracted by a standard phenol-chloroform protocol. Experimental samples and standard mixtures of mouse (skeletal muscle) and human DNA (hESC-ECP) were then run in a paired-qPCR assay (lightCycler; Roche, Switzerland) by an independent researcher with human- and mouse-specific primers (Table S1) to determine the percentage human cells within each experimental sample. As previously described,^49^ known ratios of mouse and hESC-ECP DNA were quantified by qPCR using species-specific primers (Table S1) and amplification ratios of human:mouse were generated to produce a standard curve (Figure S6). Positive (human- and mouse-only DNA) and negative (assay buffer) controls were also run.

#### Anti-human Mitochondria Immunofluorescence

To assess the distribution of human cells at early and late time points, mice were culled 4 hr and 21 days following LI surgery and immediately after injection of unlabeled hESC-ECP. Whole hindlimb muscle samples were fixed (10% formalin; 24–48 hr) before being dehydrated and wax embedded. Muscle sections from 9 levels within each sample, along with human liver (positive control) and mouse skeletal muscle (negative control), were selected for the detection of human cells using an anti-human mitochondria antibody. Briefly, rehydrated slides were blocked with 10% goat serum and incubated overnight (4°C) with anti-human mitochondria (1/100; MAB1273; Merck Millipore, USA). Primary antibody was detected with an anti-mouse-horseradish peroxidase (HRP)-conjugated antibody (1/500; PO447; Agilent Technologies, USA), and by TSA-Cy3 (1/50; NEL744; Perkin Elmer, USA). Slides were analyzed on an LSM710 confocal microscope (Zeiss, Germany).

### Investigation of the Therapeutic Potential of hESC-ECP in the LI Setting

The therapeutic potential of hESC-ECP was primarily assessed by determining the capacity of the cells to induce post-LI blood flow recovery in the several mouse models mentioned above.

Eight- or nine-week-old CD1 or nude (Crl:CD1-Foxn1^nu^) female mice (Charles River, UK) were randomly allocated into treatment or control groups. Type 1 (T1) diabetes mellitus was induced in 6- or 7-week-old CD1 female mice using STZ (Sigma-Aldrich, UK), as described previously.^50^ STZ was delivered intraperitoneally (i.p.) for five consecutive days (40 mg kg^−1^ in citrate buffer per day). Fourteen days after the first STZ injection, glycosuria was measured and only mice with overt glycosuria entered the protocol. Three months after the onset of hyperglycemia, mice underwent the LI procedure. Eleven- or twelve-week-old male db/db mice (BKS Cg-Dock7m +/+ Leprdb/J; Envigo, UK) were used as a type 2 diabetes model. Each of the above mouse types was used for experiments where cell administration was performed immediately after LI induction. In addition, subgroups of CD1 mice (left untouched until surgery time) received either hESC-ECP or fresh EBM-2 medium at 3 days following arterial ligation.

In addition to the longitudinal blood flow recovery analyses, the capillary density of the ischemic muscles of the different treatment and control groups was determined at necroscopy (21 days post-surgery).

#### Influence of hESC-ECP Administration on Blood Flow in Murine Hindlimb Ischemia Models

Blood flow in the ischemic and contralateral feet was sequentially analyzed (30 min and 7, 14, and 21 days after surgery) by color laser Doppler (Moor, UK) on anesthetized mice (1% isoflurane; 1.5 L/min) placed on a heating plate at 37°C to minimize temperature variations. The ratio of blood flow between the ischemic and contralateral foot was calculated to use as an index of percentage blood flow recovery. Color laser Doppler scans were quantified by two or three blinded, independent investigators.

### Capillary Density

At 21 days post-ischemia, after the last color laser Doppler analysis, mice were terminally anesthetized and the abdominal aorta was cannulated to perfuse the hindlimbs with 5 mM EDTA in PBS followed by 10% buffered formalin. The ischemic adductor muscle was harvested, stored overnight at room temperature (RT) in 4% paraformaldehyde (PFA), washed in PBS, and incubated in 30% sucrose for 24 hr before being embedded in optimal cutting temperature compound (OCT).

For analysis of capillary densities, adductor muscle sections were stained with isolectin B4 (Molecular Probes; I21411; 1:100) to identify the ECs and wheat germ agglutinin (Thermo Fisher Scientific; W32466) to stain muscle fiber. Slides were observed under a fluorescence microscope (Zeiss Z1 fluorescent microscope). 10 high-power fields were captured (×200), and the number of capillaries and muscle fibers per field were counted. Capillary density was expressed in two ways: (1) capillaries per mm^2^ of transverse muscle section and (2) capillary number to myofiber number ratio. Capillary density quantification was conducted by two investigators blinded to the treatment groups.

### Statistical Analysis

Data are presented as mean ± SEM, and n indicates the number of animals or experimental repeats that were performed, as indicated in the figure legends. Statistical analyses, power calculations, and graphical representations were performed with appropriate software (GraphPad Prism, GraphPad Software, La Jolla, CA, USA; Stata v. 13, StataCorp, College Station, TX, USA). For statistical comparison, the following tests were used as indicated in the figure legends: one-way ANOVA paired; one-way ANOVA with Tukey’s post hoc test; one-way ANOVA unpaired or paired with post hoc Dunnett’s test; Mann-Whitney U test; and Student’s t test. The interaction of the effect of treatment group and time in different tested scenarios was assessed with analysis of response profiles. Significance level was set at p < 0.05.

## Author Contributions

M.G.M., J.S., D.E.N., P.W.F.H., J.C.M., C.E., and A.H.B. contributed to the study concept and design. M.G.M., M.A.J., T.J.M., A.A.S.T., L.F., and H.L.S. conducted and/or analyzed the experiments conducted at the University of Edinburgh. J.S., M.B., E.M., and G.B. conducted and/or analyzed the experiments conducted at the University of Bristol. J.C.M. and A.C. produced all batches of hESC-ECP used in this study and conducted and analyzed the experiments characterizing the hESC-ECP. All authors participated in the drafting of the article and have given full approval of this version to be submitted.

## Conflicts of Interest

The authors declare that they have no competing financial interests.

## Supplemental Information

Document S1. Supplemental Materials and Methods, Figures S1–S12, and Table S1

Document S2. Article plus Supplemental Information
