# Supplementary material for: Robust Revascularization in Models of Limb Ischemia Using a Clinically Translatable Human Stem Cell-Derived Endothelial Cell Product
Source: Mol Ther. 2018 Mar 28;26(7):1669–84. doi: 10.1016/j.ymthe.2018.03.017 (PMC6035339; doi:10.1016/j.ymthe.2018.03.017)
Supplement: Document S1. Supplemental Materials and Methods, Figures S1–S12, and Table S1 [file mmc1.pdf]

YMTHE, Volume *26*

Supplemental Information

Robust Revascularization in Models of Limb Ischemia Using a Clinically Translatable Human Stem Cell-Derived Endothelial Cell Product

Mark G. MacAskill, Jaimy Saif, Alison Condie, Maurits A. Jansen, Thomas J. MacGillivray, Adriana A.S. Tavares, Lucija Fleisinger, Helen L. Spencer, Marie Besnier, Ernesto Martin, Giovanni Biglino, David E. Newby, Patrick W.F. Hadoke, Joanne C. Mountford, Costanza Emanueli, and Andrew H. Baker

Supplementary Methods:

hESC-ECP Differentiation Protocol

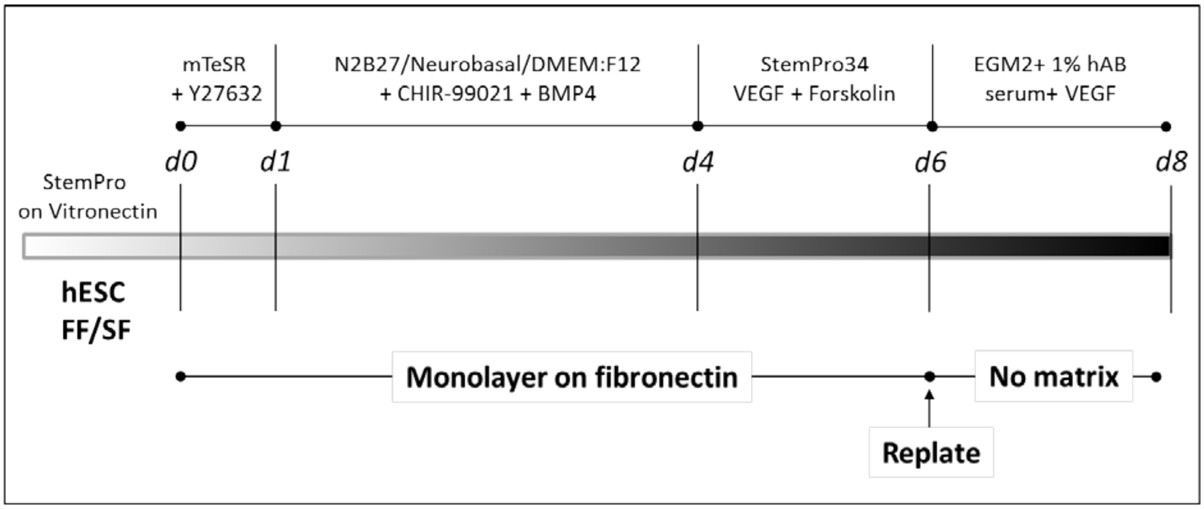

<u>Reagents</u>

GMP/Clinical/CTS Grade

StemPro EZPassage Disposable Stem Cell Passaging Tool, Life Technologies, 23181010
StemPro hESC SFM complete media, Life Technologies, A1000701
50mM beta-mercaptoethanol, Life Technologies, 31350-010
Recombinant Human FGF basic, R&D Systems, 4114-TC-01M
Recombinant Human Fibronectin GMP Protein, CF, R&D Systems, 4305-GMP
Y-27632 dihydrochloride, Tocris, 1254
StemPro Accutase, Life Technologies, A11105-01
Dulbecco's Phosphate Buffered Saline, Life Technologies, 14190-094
DMEM/F12/GlutaMAX media, Life Technologies, 10565-018
CTS Neurobasal media, Life Technologies, A13712-01
N2 CTS Life Technologies, A13707-01
B27 CTS, Life Technologies, A14867-01
GlutaMAX (100x), Life Technologies, 35050-038
Recombinant Human BMP4, GMP Protein, CF, R&D, 314-GMP
CHIR-99021, Sigma, SML-1046-5MG
StemPro-34, Life Technologies, 10639-011
Recombinant Human VEGF, GMP Protein, CF, R&D, 293-GMP
Forskolin, Sigma, F6886-10MG

Research Grade

Vitronectin (VTN-N) Recombinant Human Protein, Truncated, Life Technologies, A14700
† AB human serum from human male AB plasma, Sigma, H4522
Matrigel Basement Membrane Matrix, Growth Factor Reduced, Phenol Red Free, Corning 356231
mTeSR1, STEMCELL Technologies, 05850
Fibronectin, Sigma, F0895-5MG
Recombinant human BMP4, R&D Systems, 314-BP

Recombinant human VEGF, CF, R&D Systems, 293-VE/CF
*EBM-2, Lonza, CC3156
*EGM-2 single quots, Lonza, CC4176

† *Human AB serum is now produced under GMP by SNBTS and has been used as a direct replacement for research grade in protocol producing ATMPS.*
* *EGM2 and EBM2 from Lonza are not listed as GMP grade, but are made to the highest research use standards (see* http://www.lonza.com/about-lonza/global-citizenship/quality/quality-system.aspx). Also, *GMP certified EGM2 is available as a custom order. SNBTS have previously used this product (omitting the FCS component) as a direct replacement for the research grade reagent.*

FACS antibodies
PE Mouse anti-Human CD144, BD Biosciences, 580410
PE Mouse IgG1, κ Isotype Control, BD Biosciences, 555749
APC Anti-Human CD31, eBioscience, 17-0319-42
APC Mouse IgG1 K Isotype Control, eBioscience, 17-4714-42

PE Mouse anti-Human TRA-1-81 Antigen, BD Biosciences, 560161
PE Mouse IgM, κ Isotype Control, BD Biosciences, 555584
Alexa Fluor® 647 Mouse anti-SSEA-4, BD Biosciences, 560796
Alexa Fluor® 647 Mouse IgG3, κ Isotype Control, BD Biosciences, 560803

PE Rat anti-SSEA-3, BD Biosciences, 560237
PE Rat IgM, κ Isotype Control, BD Biosciences, 553943
Alexa Fluor® 647 Mouse anti-Human TRA-1-60 Antigen, BD Biosciences, 560850
Alexa Fluor® 647 Mouse IgM, κ Isotype Control, BD Biosciences, 560806
DRAQ7, Abcam, ab109202

Reagent Preparation

N2B27 Medium: 250ml DMEM/F12/GlutaMAX medium + 250ml CTS Neurobasal medium + 2.5mls GlutaMAX (100x) + 10ml CTS B27 + 5ml CTS N2 + 0.5ml β-Mercaptoethanol. Sterile filter 0.22 um, store at 4°C up to 1 month.

BMP4 (50μg/ml) stock: Resuspend 1mg of rhBMP4, GMP grade, carrier free (CF) in 20ml of 4mM HCl. Store at-20°C for up to a year.

CHIR-99021 (10mM) stock: Resuspend 5mg of CHIR-99021 in 1.07ml DMSO. Aliquot and store at -20°C for a year.

StemPro-34 Medium: 500ml StemPro-34 medium + 5ml Glutamax (100x) + 13ml StemPro-34 supplement. Store at 4°C up to 1 month

VEGF165 (50µg/ml) stock: Dissolve 1mg of rhVEGF165, GMP grade, carrier free (CF) in 20ml PBS. Store at -20°C for a year.

Forskolin (10mM) stock: Dissolve 10mg of forskolin in 2.44ml DMSO. Store at -20°C for a month.

EGM-2 Medium: 500ml EBM-2 medium + single quots of EGM-2 supplements omitting both VEGF +FBS (i.e. add only supplement quots of hydrocortisone, rhFGF-B, R3-IGF1, ascorbic acid, rhEGF, GA-1000 and heparin).

Differentiation protocol

The protocol below is to differentiate cells in oneT25 flask (Corning, 430639). If performing differentiation in wells or larger flasks plate adjust media volume accordingly to maintain the volume/cm^2 surface area ratio.

Maintenance of hESCs

Human embryonic stem cells are cultured on vitronectin coated wells in StemPro complete medium with 20ng/ml bFGF. Cultures are passaged when confluent (every 6-7 days) using EZPassage disposable stem cell passaging tools.

Day 0: Plating of hESCs

1. Coat a T25 (Corning, 430639) with 300µl 250µg/ml fibronectin (3µg/cm^2) using a cell scraper (Corning, 3010). Incubate for I hour at room temperature, on a shaking platform.
2. Aspirate growth medium from an 80% - 100% confluent well of hESCs. Wash with DPBS (Ca^{2+} and Mg^{2+} free). Add 1ml pre-warmed Accutase and incubate 3-7 min at 37°C. Check cells' detachment under a microscope.
3. Add 1ml pre-warmed mTeSR1 + Rock Inhibitor (final conc. 10µM). Pipette gently up and down to mix and transfer cells to a 15ml Falcon tube
4. Centrifuge the suspension at 1200 rpm (310g) for 3mins.
5. Aspirate supernatant and resuspend the cell pellet in 2ml pre-warmed mTeSR1+ ROCK-Inhibitor (final conc. 10µM).
6. Count the cells and seed the cells at 20,000-40,000 hESCs per cm^2. This should be optimised for each cell line and batch of cells.
7. Incubate the cells at 37°C, 5% CO_2 overnight.

Day 1-3: Lateral mesoderm induction

1. Replace media with pre-warmed N2B27 medium supplemented with 7µM CHIR-99021 + 25ng/ml rhBMP4 (7.5ml/T25). Cells are left for 3 days without medium change.

Day 4 and 5: Endothelial induction

1. Replace Media with 7.5ml/T25StemPro-34 SFM medium supplemented with 200ng/ml VEGF and 2µM forskolin. Change medium every day.

Day 6: Endothelial cell replating

1. Aspirate medium and wash cells with DPBS (Ca^{2+} and Mg^{2+} free). Add 2ml pre-warmed Tryple Express and incubate 3-7 min at 37°C. Check cell detachment under a microscope.

2. Transfer dissociated cells to a 15ml Falcon tube, rinse flask with 2 x 3ml DPBS and add to tube. Fill to the top with DPBS (Ca^{2+} and Mg^{2+} free) and pipette gently up and down to mix.
3. Centrifuge at 310g for 3mins at RT.
4. Aspirate supernatant and resuspend the cell pellet in 2ml pre-warmed EGM-2 media (EBM-2 + EGM2 single quots *omitting both VEGF and FBS quots*) +50ng/ml VEGF +1% hAB serum
5. Count the cells and seed at 40,000 cells per cm^2 ($1x10^6$ cells in a T25) in EGM-2 media +50ng/ml VEGF + 1% hAB serum

Day 7: Feed
Replace media with fresh EGM-2 media +50ng/ml VEGF +1% hAB serum

Day 8: Harvest endothelial cells
1. Aspirate medium and wash cells with DPBS (Ca^{2+} and Mg^{2+} free). Add 2ml pre-warmed Tryple Express and incubate 3-7 min at 37°C. Check cells' detachment under a microscope.
2. Transfer dissociated cells to a 15ml Falcon tube, rinse flask with 2 x 3ml DPBS and add to tube. Fill to the top with DPBS (Ca^{2+} and Mg^{2+} free) and pipette gently up and down to mix.
3. Centrifuge at 310g for 3mins.
4. Aspirate supernatant and resuspend the cell pellet in 0.5ml pre-warmed EGM-2 media (EBM-2 + EGM-2 single quots *omitting both VEGF and FBS quots*) +50ng/ml VEGF +1% hAB serum.
5. Count the cells.

Day 8: Matrigel tubule assay
1. The day before seeding the cells, slowly thaw the Matrigel on ice in a 4°C fridge overnight. Also place flat bottomed 96 well plate (Corning, 3596) and 200µl pipette tips in a -20°C freezer overnight.
2. The next day, working in a laminar flow hood add 50µl Matrigel per well required using chilled pipette tips, avoiding bubbles and working quickly.
3. Tap the sides of the plate gently to ensure the gel has spread evenly. Place the plate in a 37° C incubator for 30 minutes to allow polymerisation.
4. Adjust the d8 cell suspension (in EGM-2 +50ng/ml VEGF +1% hAB serum) to $2x10^5$ cells/ml and add 100µl to triplicate wells containing matrigel (20,000/well).
5. Image the tubules on a brightfield microscope at 3 hours and 6 hours.

Flow Cytometry (Day 0, 6 and 8).
Antibody staining for flow cytometry should be carried out on d0 (hESCs), d6 and d8 cells. Briefly, stain 1x105 cells in PBS/tube for 30 mins at 4°C in the dark, wash with 1 ml DPBS at 310g, resuspend in 300µl PBS and analyse immediately.

Cells should be stained with protein matched isotype controls or the antibody combinations below. Flow cytometric analysis in this study was carried out on a BDFACS CANTO II. The gating plan is shown in Figure X. Live cells should be gated, then doublets excluded. Quadrants/region gates should be set using the matched isotype controls (as detailed above).

d0
Tube 1: TRA-1-81 -PE, SSEA-4- Alexa Fluor® 647
Tube 2: SSEA-3- PE, TRA-1-60- Alexa Fluor® 647

d6
Tube 1: TRA-1-81-PE, SSEA-4- Alexa Fluor® 647
Tube 2: SSEA-3-PE, TRA-1-60- Alexa Fluor® 647
Tube 3. CD144-PE, CD31-APC

d8
Tube 1: TRA-1-81-PE, SSEA-4- Alexa Fluor® 647
Tube 2: SSEA-3-PE, TRA-1-60- Alexa Fluor® 647
Tube 3: CD144-PE, CD31-APC
Tube 4: CD144-PE, DRAQ7

Assessment of the impact of transportation on hESC-ECP phenotype and function

The efficacy and engraftment studies were performed after courier shipment of hESC-ECP from Glasgow to Edinburgh/Bristol (up to 7 hour delivery time). To investigate the impact of shipping on the hESC-ECP studies were performed simulating transport by storing hESC-ECP batches at room temperature for 7h on d7 of differentiation. Flow cytometry for $CD31/CD144^{+/+}$ was performed to confirm the EC phenotype and Matrigel angiogenic assays were undertaken to confirm functional capacity of hESC-ECP kept under normal culture conditions or exposed to shipping mimicking conditions. Additionally, investigators based at UoE and UoB periodically performed Matrigel tubule formation assays following delivery of cells.

Longitudinal MRI tracking of hESC-ECP

All MRI experiments were performed using a 7 Tesla horizontal bore NMR spectrometer (Agilent Technologies, Yarnton, UK), equipped with a high-performance gradient insert (60 mm inner diameter), maximum gradient strength 1000 mT/m. The mice were anesthetized with 1.8 % isofluorane in oxygen/air (50/50, 1 L/min) and placed in a cradle (Rapid Biomedical GmbH, Rimpar, Germany), their temperature was maintained at 37°C with a heat fan. A 39-mm diameter birdcage volume coil (Rapid Biomedical GmbH, Rimpar, Germany) was used for radio frequency transmission and signal reception. For T2* mapping and calculation of T2* relaxation times in the limbs, image acquisition used a gradient echo, multiple echo, pulse sequence of twenty images weighted in T2*; TE = 0.93 – 22.53 ms, TR = 100 ms, 40 x 40 mm field of view, 192 × 192 acquisition matrix (in-plane resolution = 0.208 × 0.208 mm) and 30° flip angle. Slice thickness was 1 mm with 8 signal averages. For T2* mapping and calculation of T2* relaxation times in the organs, seven respiratory-gated gradient echo images were acquired with TEs varying from 1.8 to 15 ms, TR = 60 ms, 35 x 35 mm field of view, 128 × 128 acquisition matrix (in-plane resolution = 0.273 × 0.273 mm) and 20° flip angle. Slice thickness was 1 mm with 2 signal averages. Scans were performed at baseline (prior to administration of hESC-ECP), and 1, 7, 14 and 21 days post-administration. T2*-weighted multi-gradient-echo images for each scan were combined to generate a T2* map, in which the data represented the T2* value ($S(t)=S(0)\exp-(t/T2^*)$) for each voxel. This was achieved using in-house software called MAPPED (version 3.4, University of Edinburgh)

that was developed in Matlab (Mathworks, USA). The T2* value is the gradient of the line of best fit to the natural logarithm of the exponential decay of signal intensity with time. In the presence of SPIO, the signal decays more rapidly because of local field inhomogeneities and the T2* value is reduced. A 3×3 voxel Gaussian filter was applied to the individual echoes to reduce noise. Using a robust multilinear regression to fit a line of best fit to the log of the data, T2* was obtained. This fitting process is less influenced by outliers than a least-squares approach. An experimentally determined threshold for the coefficient of determination was used to exclude data that did not have an acceptable fit when the log of signal intensity (SI) was plotted against echo time.

Iron Stain (Prussian Blue)
A histological iron stain (Abcam, uk) was used to visualise SPIO uptake in hESC-ECP. Briefly, d7 hESC-ECP were seeded onto glass coverslips and labelled overnight with SPIO. Coverslips were then fixed for 10min with 2% paraformaldehyde, washed with PBS and incubated with 1:1 of Reagent A and B of the iron stain for 3 min. The coverslips were then washed and counterstained with nuclear fast red before being mounted and imaged by brightfield microscopy.

Ferrozine Assay
A adapted ferrozine assay (Riemer et al., 2004) was performed to quantify the level of SPIO uptake within labelled hESC-ECP. Briefly, labelled cells were washed with PBS before being lifted, counted and aliquoted at $5x10^4$ cells per 1.5ml Eppendorf (all samples performed in triplicate). The cells were then pelleted before adding 50µl of 1.2M HCl. Iron standards were prepared (3-0.05µg Fe, Cat.No.16596, Sigma Aldrich, UK) in 100µl 0.6M HCl. All experimental and standards tubes were incubated at 65°C for 2h, after which 50µl of dH_2O was added to the experimental standards. 30µl of ferrozine reagent (6.5 mM ferrozine, 6.5 mM neocuproine, 2.5 M ammonium acetate, and 1 M ascorbic acid dissolved in dH_2O) was then added to all tubes and incubated for 30 min. The absorbance was then read at 570nm, a standard curve created, and the iron content per cell calculated.

MTT Assay
An MTT assay (Sigma Aldrich, UK) was used to determine the % viability of labelled cells, according to the manufacturer's instructions. Briefly, $1.5x10^4$ hESC-ECP were seeded per well of a 96 well plate (in triplicate) on d7, followed by SPIO labelling overnight. On d8, 10% MTT reagent was then added to each well and incubated for 4 hours at 37°C. Culture medium was removed from each well and 100µl of DMSO was added. Absorbance was measured 570 nm, with background at 690nm subtracted from the final result. % Viability was calculated relative to control, untreated cells. 0.1% Triton X-100 (Sigma Aldrich, UK), was added to the cells as a cytotoxic control.

Lactate Dehydrogenase Assay
A lactate dehydrogenase assay (Thermo Fisher Scientific, USA) was used to determine the % cytotoxicity of labelled cells, according to the manufacturer's instructions. Briefly, $1.5x10^4$ hESC-ECP were seeded per well of a 96 well plate (in triplicate) on d7, followed by SPIO

labelling overnight. On d8, 50µl of culture medium was removed from each well and incubated with the LDH reaction mixture for 30 min at room temperature. 50µl of stop solution was added to each well and the absorbance was then measured at 570 nm, with background at 690nm subtracted from the final result. The % cytotoxicity was calculated according to the manufacturer's instructions. 0.1% Triton X-100 (Sigma Aldrich, UK), was added to the cells as a cytotoxic control.

Transwell Migration

Labelled d8 hESC-ECP were transferred to EBM-2 containing 0.1% FCS for 4 hours prior to harvest for the migration assay. 6.5mm, 8µm pore size transwell inserts were precoated with 10µg/ml fibronectin and 0.1% gelatin (Sigma Aldrich) and added to a 24 well plate which contained 400µl EBM-2 with 100ng/ml VEGFA (Peprotechc, USA). Labelled hESC-ECP, suspended in EBM-2 with 0.1% bovine serum albumin (Sigma Aldrich, UK) were added to the top of the chamber ($5x10^4$ in 200µl) and incubated for 4 hour under culture conditions. hESC-ECP which had not migrated were then removed from the top of the chamber using a cotton swab before the remaining cells were fixed in cold methanol (20°C, 5min). Following two PBS washes, cells were counterstained with DAPI (Sigma Aldrich, UK) and the filter cut and mounted for visualisation of migrated cells. The mean number of hESC-ECP over 5 fields of view was calculated for each filter to determine the level of migration.

Ac-LDL uptake

An acetylated LDL uptake assay was performed to assess endothelial function as previously described (Drebert et al., 2016).

Tubule formation

Tubule formation on Matrigel was performed as detailed in the main manuscript.

Haemocytometer Assessed Proliferation and Viability

To assess the impact of ^{18}F-FLT labelling on viability and proliferation, labelled cells were subcultured for 5 days in order to detect the long lasting effects of DNA damage. Viable/ non-viable labelled cells were counted using a neubauer haemocytometer and trypan blue (0.4%, Sigma Aldrich, UK). The % viability and proliferation were quantified relative to control, untreated cells.

FACs cell cycle analysis

To assess the impact of ^{18}F-FLT labelling on cell cycle, labelled cells were subcultured for 5 days in order to detect the long lasting effects of DNA damage. Cell cycle analysis was carried out as previously described (MacAskill et al., 2017).

Transcript expression analysis

hESC-EC populations were harvested on day 8 of differentiation and separated using MACS beads conjugated with CD144 (Miltenyi Biotech, Germany). After separation, samples of the positive and negative fractions were analysed for expression of CD144 and CD31 by flow

cytometry. RNA was prepared from both the CD144+ and CD144- fractions from 3 independent experiments.

Total RNA from cell populations was isolated using the miRNeasy kit (Qiagen, U.K). For gene analysis, RNA was transcribed using random primers. RNA was transcribed following manufacturer's instructions. Human mRNAs were analysed using TaqMan assays (Life Technologies, UK). Ubiquitin C (UBC) was used for normalization (Life Technologies, Paisley, UK) (**Table below**). Quantitative PCR reactions were performed in technical duplicates/triplicates with the QuantStudio 5 Real-time PCR system (Life Technologies, UK). Quantitative expression was calculated using the 2-(ΔΔCT) (RQ) method.

GENE ID	REFERENCE
Angpt1	Hs00919202_m1
Angpt2	Hs00169867_m1
FGF-2	Hs00266645_m1
TGF-B2	Hs00234244_m1
UBC	Hs01541556_s1
VEGF-A	Hs0090055_m1
VEGF-C	Hs01099203_m1
CD31	HS01065282_m1

Supplementary Figures:

A

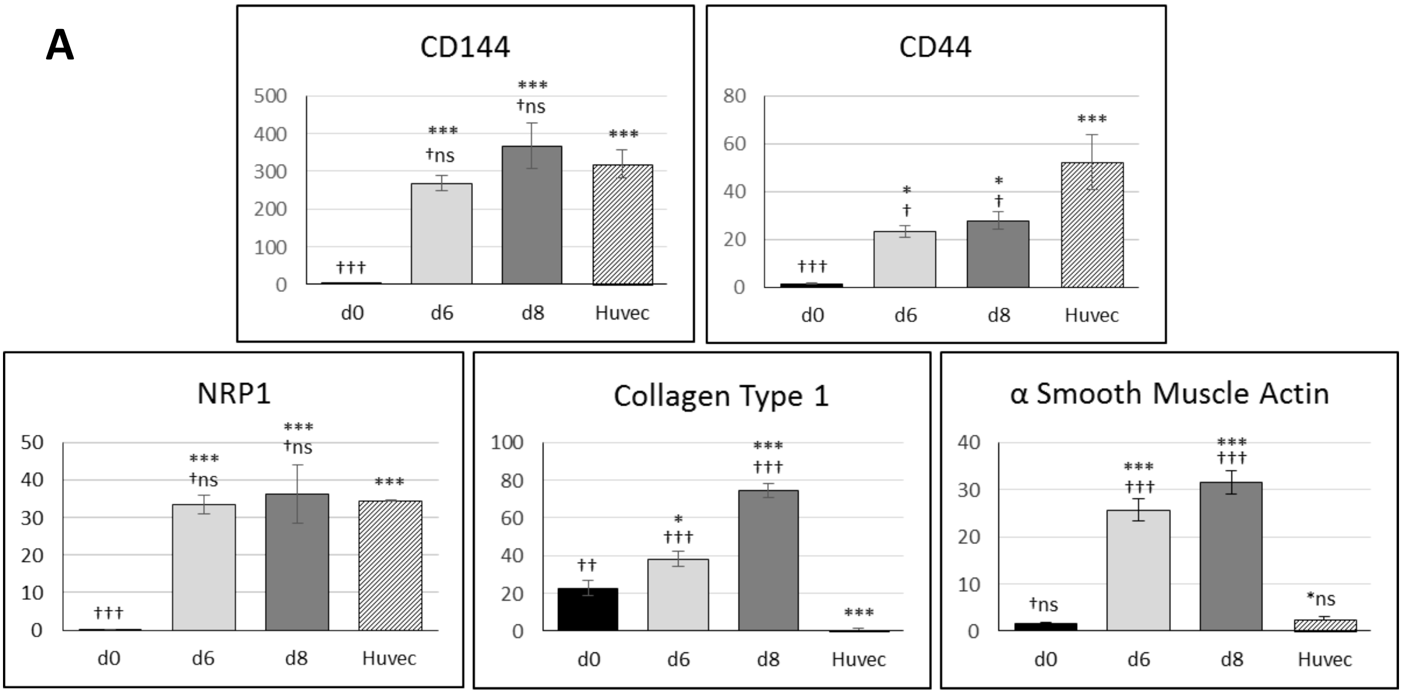

B

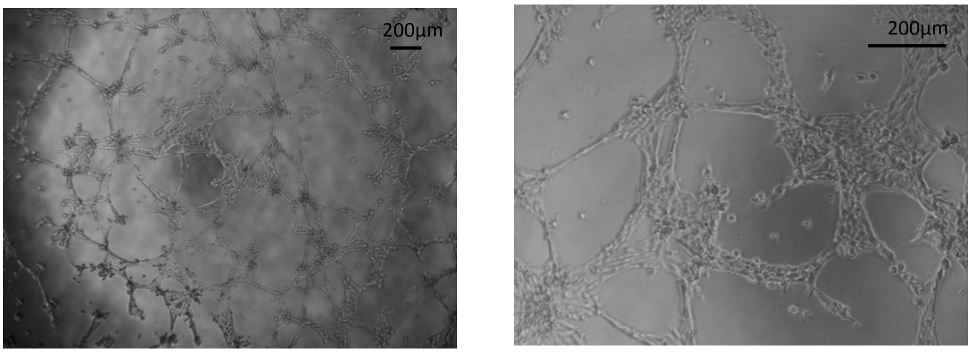

Supplementary Figure 1. Additional Characterisation of hESC-ECPs. **A**) Expression of selected pluripotent and endothelial genes in differentiated RC11 cells. Quantitative RT-PCR analysis of mRNA for additional genes during the differentiation process compared with mRNA from human umbilical vein endothelial cells (HUVEC) as a positive control. Data are shown as 2ΔCt x1000 compared to the housekeeping gene β-actin. hESC data are n=4 biological replicates assayed in triplicate, HUVEC n=3 in triplicate,*$p<0.05$, **$p\leq0.01$, ***$p\leq0.001$ significance compared with d0, and †$p<0.05$, ††$p\leq0.01$,†††$p\leq0.001$ significance compared with HUVEC using one-way ANOVA with Tukey's post-hoc test. **B)** Phase contrast microscopy pictures showing tubule formation in Matrigel after 6 hours.

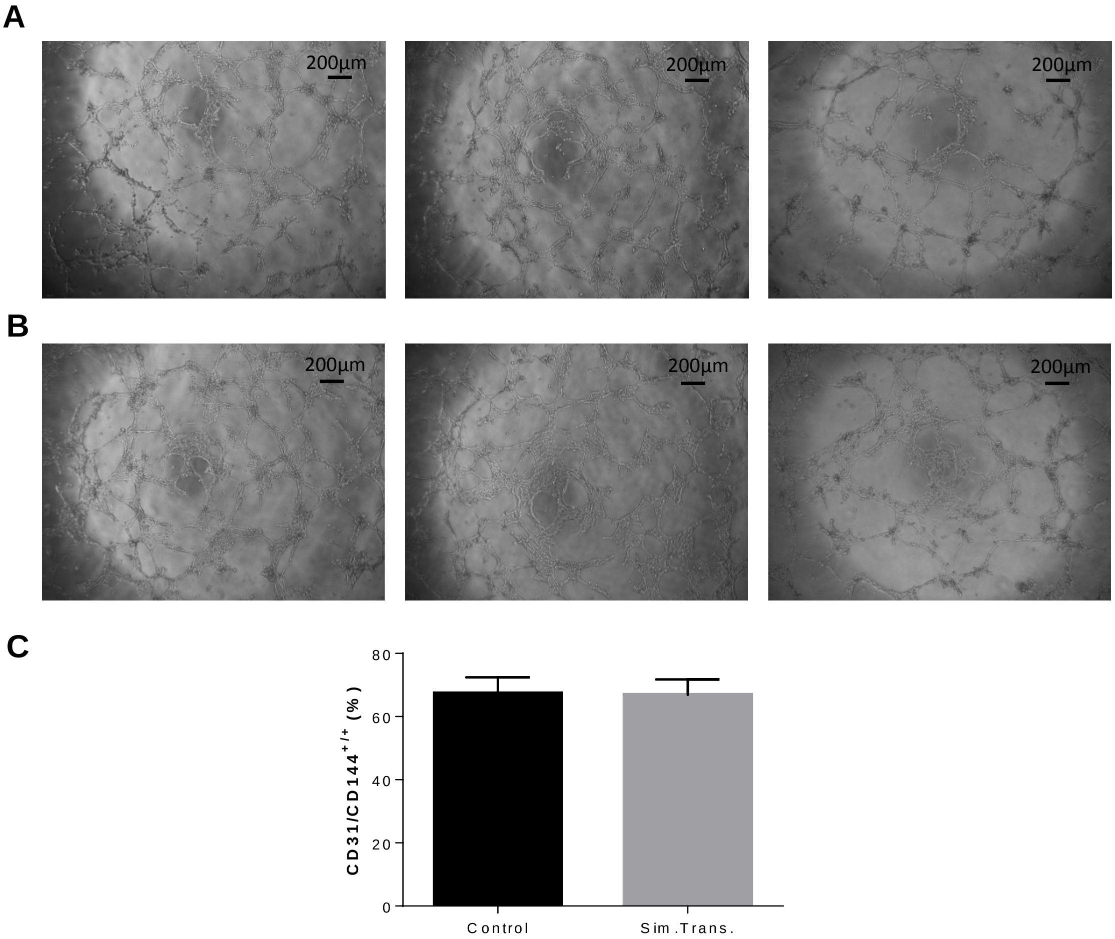

Supplementary Figure 2. Transportation of hESC-ECP at room temperature on d7 does not affect tubule formation or endothelial marker expression at d8. A) d8 example images from three hESC-ECP batches which remained in culture conditions on d7 showing good tubule formation on matrigel. **B)** d8 example images from three hESC-ECP batches which were stored at room temperature for 7h on d7 to simulate transport showing good tubule formation on matrigel. **C)** FACs quantification of $CD31/CD144^{+/+}$ expression in control and simulated transport (Sim.Trans.) hESC-ECP showing no difference, n=3.

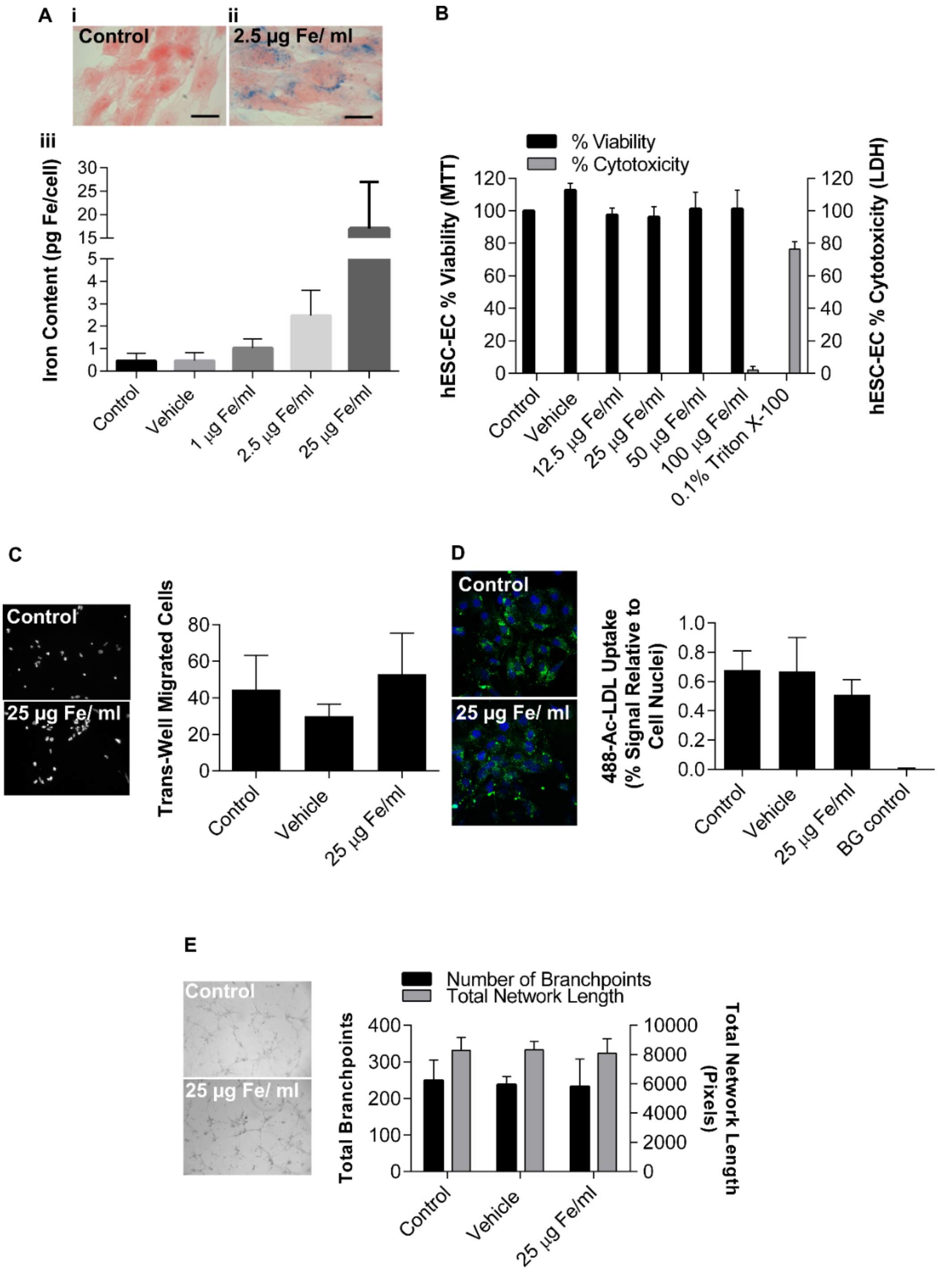

Supplementary Figure 3. SPIO-labelling of hESC-ECP does not affect cell viability or function. hESC-ECP were incubated overnight with several concentrations of SPIO and intracellular iron content was confirmed by Prussian Blue stain for A**i)** control or A**ii)** SPIO (2.5 µg Fe/ml)-treated cells. Intracellular iron content was quantified by A**iii)** Ferrozine assay, n=3. hESC-ECP viability and cytotoxicity were assessed by **B)** MTT and lactate dehydrogenase assay following SPIO labelling, n=3. The effect of SPIO labelling on endothelial function was assessed by; **C)** trans-well migration towards VEGF (100ng/ml), n=3, **D)** Ac-LDL uptake, n=3 and **E)** tubule formation on Matrigel, n=4. All data represent mean ± S.E.M.; comparisons were made vs. untreated control using a one-way ANOVA with

post-hoc Dunnett's test. No significant differences in the function of hESC-ECP were detected between control and treated cells.

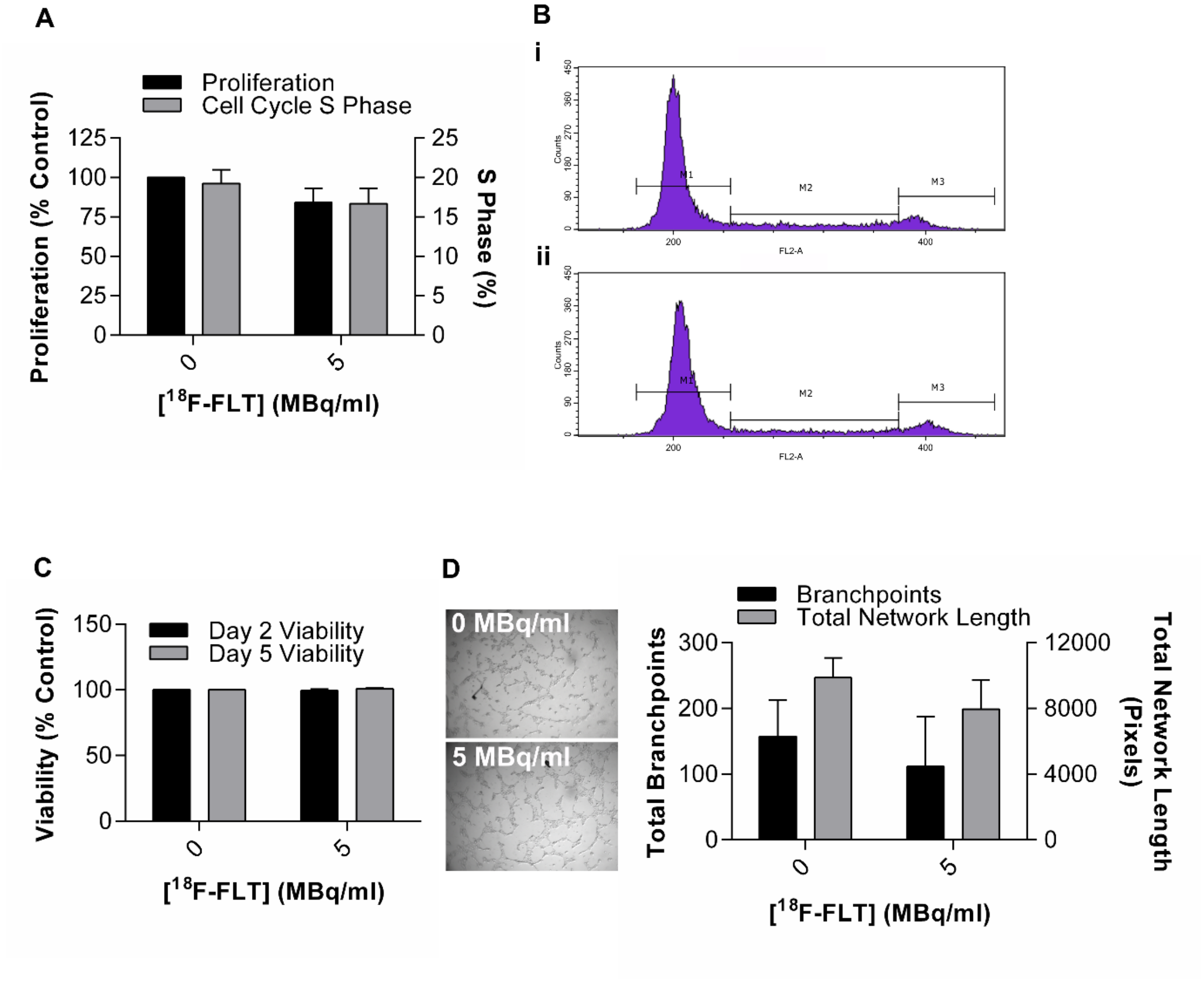

Supplementary Figure 4. ^{18}F-FLT-labelling of hESC-ECP does not affect cell viability, proliferation or function. Following incubation (1hr) with ^{18}F-FLT or control medium, cells were re-plated and assessed 5 days post labelling. **A)** hESC-ECP proliferation calculated by total cell counts (n=4) and quantification of cell cycle S phase (n=3) assessed by propidium iodide, with example FACs cell cycle profiles for **Bi)** control and **Bii)** ^{18}F-FLT labelled hESC-ECP. **C)** Viability of hESC-ECP assessed by trypan blue exclusion 2 days (n=3) and 5 days (n=4) post labelling. **D)** Tubule formation on Matrigel, n=4. All data represent mean ± S.E.M.; comparisons between control and treated hESC-ECP were made using Student's t-test. No significant differences were detected.

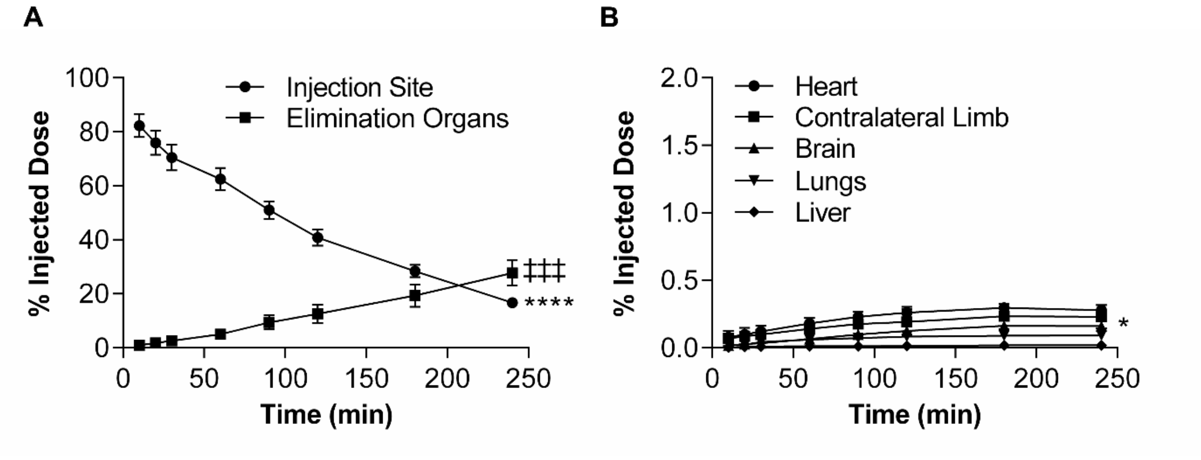

Supplementary Figure 5. Dynamic PET time activity curves show minimal accumulation of signal outside the injection site and elimination organs. Decay-corrected quantification of radiolabel at **A)** injection site and elimination organs (Kidney and Bladder), n=5, ****=p≤0.0001 one-way ANOVA paired for signal over time, ‡‡‡= p≤0.001 one-way ANOVA paired for signal over time and **B)** sites of interest for radiolabel/cell uptake, n=6, *p<0.05 for heart, contralateral limb, brain, lungs and liver, one-way ANOVA paired for signal over time.

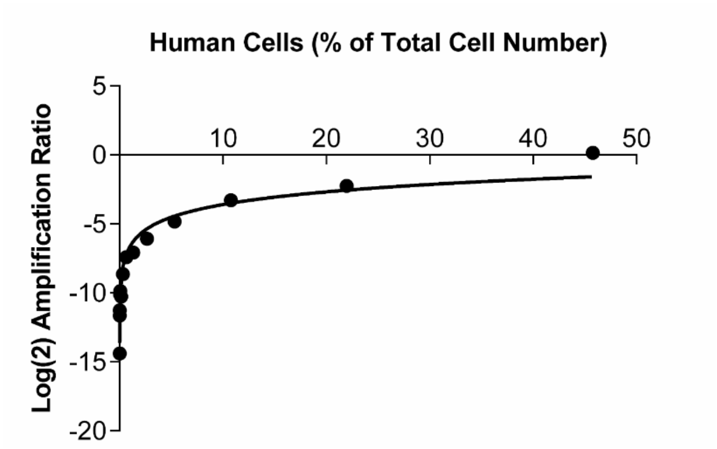

Supplementary Figure 6. Standard curve used in PCR quantification of % Human cells present within murine ischemic hind-limbs. Non-linear curve fitting of Log(2) amplification ratio (Human:Mouse ct value) and % Human cells calculated from standards with known proportions of human and mouse DNA, performed in triplicate.

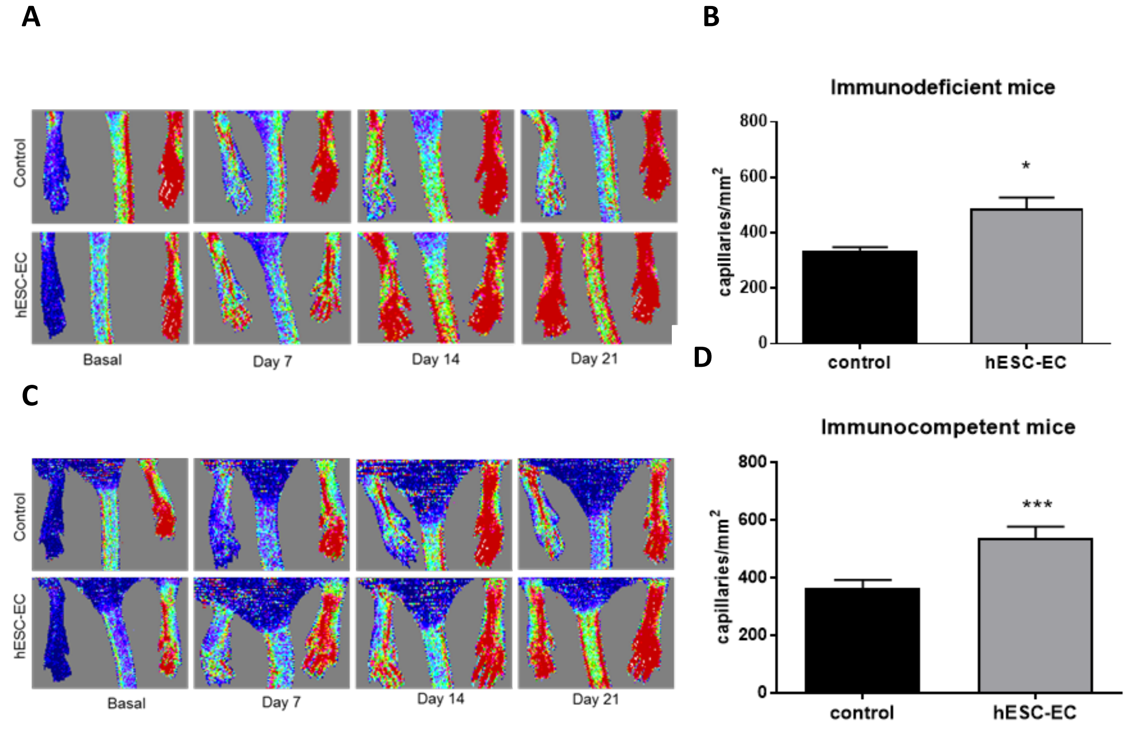

Supplementary Figure 7. Efficacy studies in immunodeficient (Crl:CD1-Foxn1^{nu}) and immunocompetent (CD1) mice with acute ischemia at cell transplantation. Representative color laser Doppler blood perfusion images of ischemic limbs of immunodeficient (**A**) and immunocompetent(**C**) mice. Immediately post-surgery, the ischemic muscles were injected with $1x10^6$ hESC-ECP or vehicle (EBM-2) as control. Top rows show longitudinal (0, 7, 14 and 21 days after surgery) scans of control group mouse (treated with fresh EBM-2) and bottom rows show scans of hESC-ECP-treated mice. Capillary density (expressed as capillary per mm^2 in immunodeficient mice n=7, p value <0.05, Mann Whitney U test (**B**) and immunocompetent mice (**D**) is shown; n=12, p value <0.05, Mann Whitney U test.

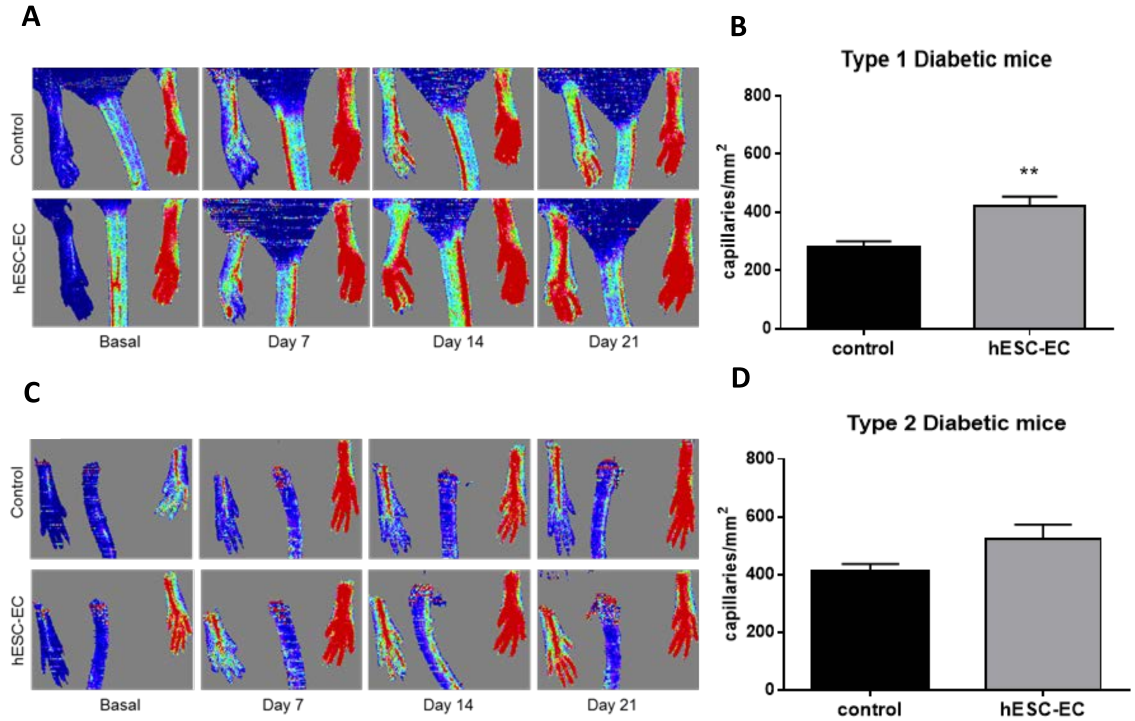

Supplementary Figure 8. Efficacy study in type 1 and type 2 diabetic mice with acute ischemia at cell transplantation. Representative colour laser Doppler blood perfusion images of ischemic limbs of **A)** type 1 diabetic and **C)** type 2 diabetic mice. Immediately post-surgery, the ischemic muscles were injected with $1x10^6$ hESC-ECP or vehicle (EBM-2) as control. Top rows show longitudinal (0, 7, 14 and 21 days after surgery) scans of control group mice (treated with fresh EBM-2) and bottom rows show scans of hESC-ECP-treated mice. Capillary density expressed as capillary per mm^2 in **B)** type 1 diabetic mice and **D)** type 2 diabetic mice is shown; n=7, p value <0.05, Mann Whitney U test.

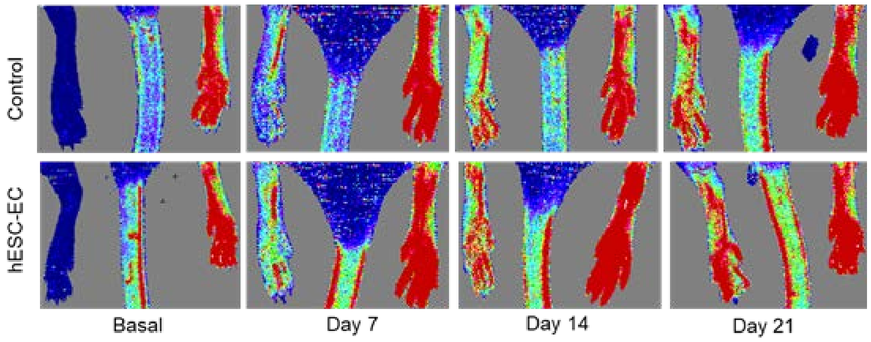

Supplementary Figure 9. Efficacy study in immunocompetent, non-diabetic CD1 mice with established ischemia at cell transplantation. Representative colour laser Doppler blood perfusion images of ischemic limbs of CD1 mice that received hESC-ECP 3 days post-ischemia induction. Top rows show longitudinal (0, 7, 14 and 21 days after surgery) scans of control mice (treated with fresh EBM-2) and bottom rows show scans of hESC-ECP-treated mice.

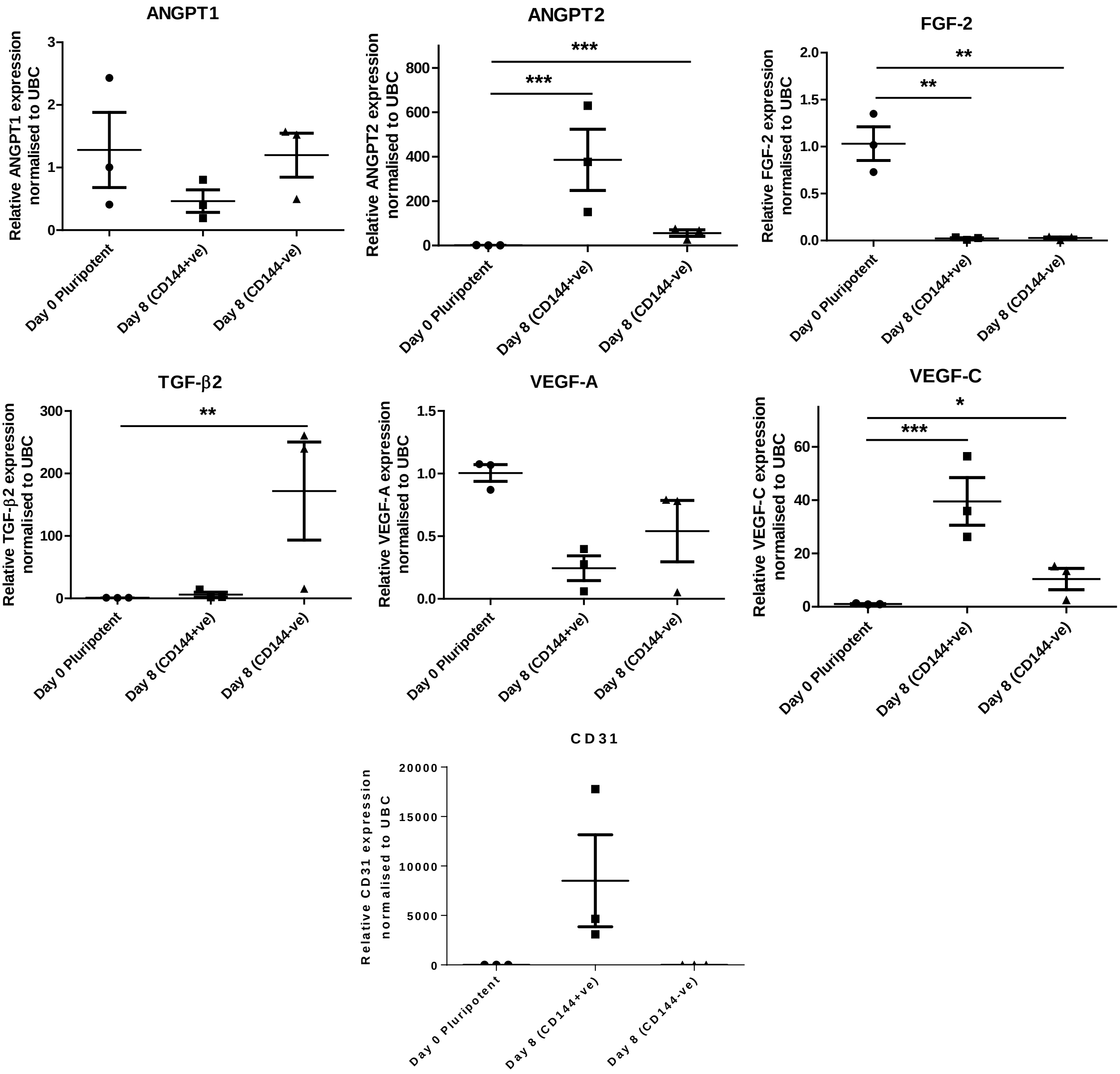

Supplemental Figure 10. Assessment of angiogenesis-associated genes in the CD144+ve and -ve populations within hESC-ECP. On day 8 of differentiation, hESC-ECP were sorted by MACs conjugated with CD144 and assessed for angiogenic signatures by qPCR. The results are representative of three independent hESC-EC productions and expressed as the mean ± S.E.M., *= $p<0.05$, **= $p<0.01$ and ***= $p<0.001$ vs. Day 0 using a one-way ANOVA with post-hoc Dunnett's test.

A

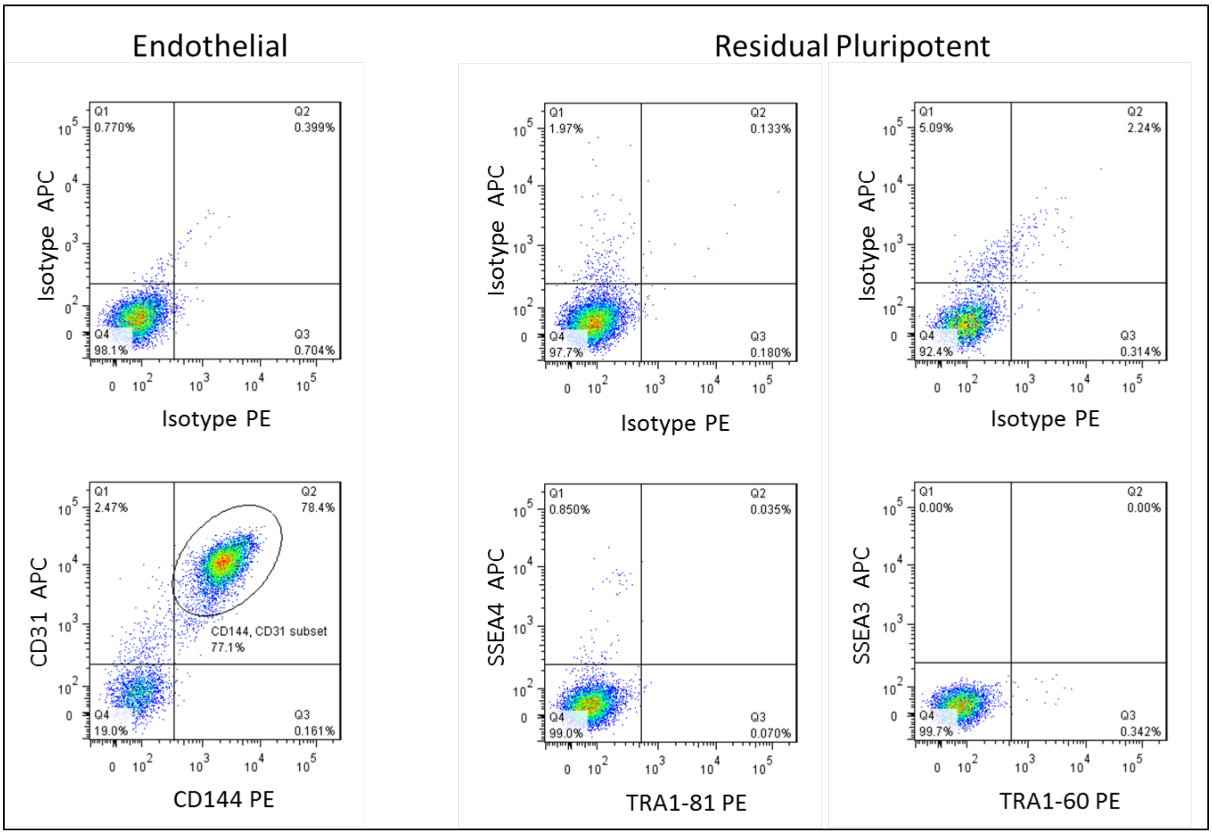

B

Experiment	d0 Cells (Passage)	Fold Change d0 to d8	d8 % CD144/CD31 double positives	d8 % SSEA4/TRA1-81	d8 % SSEA3/TRA1-60	d8 viability (%)
ACEND152	H9 (P39)	4.00	70.8	0.008	0.000	95.0
ACEND153	H9 (P40)	5.44	77.1	0.000	0.000	94.0
ACEND154	H9 (P41)	7.65	87.3	0.000	0.000	94.0
**Average n=3**		**5.70**	**78.40**	**0.003**	**0.000**	**94.33**
**± SEM**		**1.06**	**4.81**	**0.003**	**0.000**	**0.33**

Supplemental Figure 11. Endothelial differentiation of H9 hESC.
In additional to the clinical grade RC11 line, we also used H9 cells (WiCell, Wisconsin, USA) in the differentiation protocol. Performance of this cell line was comparable to RC11 with similar expansion in cell number and % CD144/CD31+ cells generated by d8. Differentiated H9 cells analysed on day 8 of the protocol predominantly co-express the endothelial markers CD31 and CD144 with few, if any, detectable residual pluripotent hESC. **A)** Representative flow cytometric analysis (from Experiment ACEND 153) for the endothelial (left panels) and pluripotent markers (middle and right panels) with the appropriate isotype controls. Cells were pre-gated for viable cells (FSC/SSC; 10,000 events) and doublet exclusion (FSC-A/FSC-H). **B)** Day 8 H9 hESC-ECP characteristics including the fold change in viable cell number from d0-d8 and marker profile after correction for isotype values, n=3 replicates, data expressed as mean ± SEM.

A

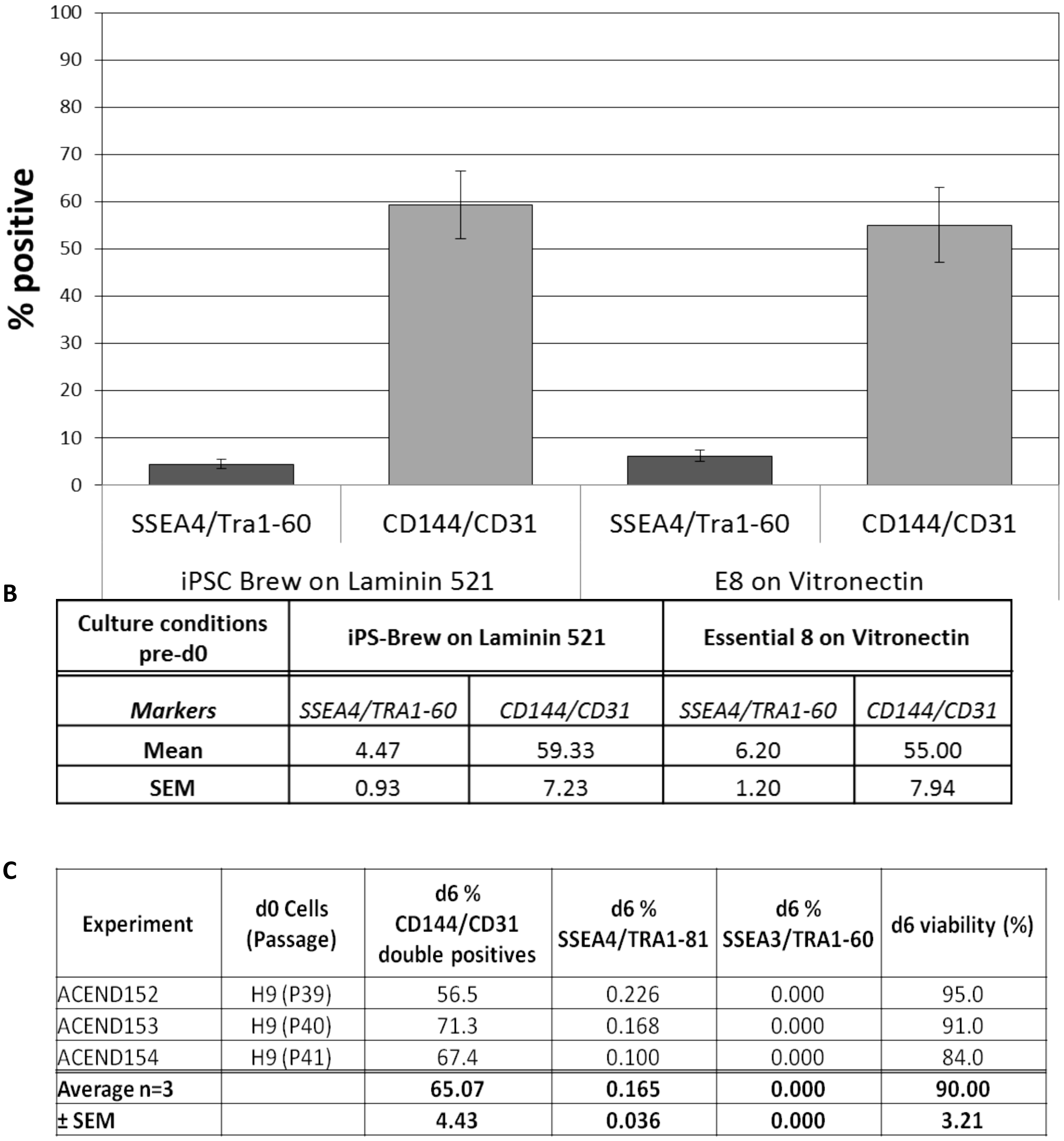

B

Culture conditions pre-d0	iPS-Brew on Laminin 521		Essential 8 on Vitronectin	
Markers	*SSEA4/TRA1-60*	*CD144/CD31*	*SSEA4/TRA1-60*	*CD144/CD31*
**Mean**	4.47	59.33	6.20	55.00
**SEM**	0.93	7.23	1.20	7.94

C

Experiment	d0 Cells (Passage)	d6 % CD144/CD31 double positives	d6 % SSEA4/TRA1-81	d6 % SSEA3/TRA1-60	d6 viability (%)
ACEND152	H9 (P39)	56.5	0.226	0.000	95.0
ACEND153	H9 (P40)	71.3	0.168	0.000	91.0
ACEND154	H9 (P41)	67.4	0.100	0.000	84.0
**Average n=3**		**65.07**	**0.165**	**0.000**	**90.00**
**± SEM**		**4.43**	**0.036**	**0.000**	**3.21**

Supplemental Figure 12. Endothelial differentiation of NAS2 iPSC.
In addition to RC11 and H9 hESC lines, we also used NAS2 cells (Devine et al., 2011) (a kind gift from Tilo Kunath Edinburgh University, Edinburgh, UK), in the differentiation protocol. As these cells were used for a different purpose and the differentiation was stopped at day 6, however, we did test NAS2 stocks that had been grown in 2 additional culture systems that are available at GMP grade, either iPS-Brew XF medium (Miltenyi Biotech) on laminin 521 (Biolamina) or in Essential 8 on rh Vitronectin (Life Technologies). Performance of this cell line in either medium/matrix system, was comparable to RC11 and H9, generating ≥55% CD144/CD31+ cells by d6. **A)** Data from flow cytometric analysis, after correction for the appropriate isotype controls. **B)** Tabulated data from part A shown as mean % positive ± SEM on day 6. n=3 replicates. **C)** Data for H9 hESC differentiations on d6 of differentiation (d8 data for the same experiments shown in Supplemental Figure 11) for comparison to d6 NAS2 data.

Species Specific Primers		
	Forward	Reverse
**Human**	5'-ctgttttgtggcttgttcag-3'	5'-aggaaaccttccctcctcta-3'
**Mouse**	5'-ttggttgagaagcagaaaca-3'	5'-cacacagtcaagttcccaaa-3'

Supplementary Table 1. Species specific primers used in PCR detection of human and mouse DNA
